# Characterization of *Portulaca oleracea* Whole Plant: Evaluating Antioxidant, Anticancer, Antibacterial, and Antiviral Activities and Application as Quality Enhancer in Yogurt

**DOI:** 10.3390/molecules28155859

**Published:** 2023-08-03

**Authors:** Diana A. Al-Quwaie, Aminah Allohibi, Majidah Aljadani, Amira M. Alghamdi, Asmaa Ali Alharbi, Roua S. Baty, Safa H. Qahl, Ohud Saleh, Amani Osman Shakak, Fatimah S. Alqahtani, Osama S. F. Khalil, Mohamed T. El-Saadony, Ahmed M. Saad

**Affiliations:** 1 Biological Sciences Department, College of Science & Arts, King Abdulaziz University, Rabigh 21911, Saudi Arabiaaoosman@kau.edu.sa (A.O.S.); 2 Department of Chemistry, College of Sciences & Arts, King Abdulaziz University, Rabigh 21911, Saudi Arabia; maaljadani@kau.edu.sa; 3 Department of Biochemistry, Faculty of Science, King Abdulaziz University, Jeddah 21589, Saudi Arabia; amaalghamdi1@kau.edu.sa (A.M.A.); aanalharbi@kau.edu.sa (A.A.A.); 4 Department of Biotechnology, College of Science, Taif University, P.O. Box 11099, Taif 21944, Saudi Arabia; rsbaty@tu.edu.sa; 5 Department of Biology, College of Science, University of Jeddah, Jeddah 21589, Saudi Arabia; shqahal@uj.edu.sa; 6 Department of Biochemistry, College of Science, University of Jeddah, Jeddah 21959, Saudi Arabia; oasaleh@uj.edu.sa; 7 Faculty of Medical Laboratory Sciences, University of Shendi, Shendi P.O. Box 142, Sudan; 8 Department of Biology, Faculty of Sciences, University of Bisha, P.O. Box 551, Bisha 61922, Saudi Arabia; faalqahtani@ub.edu.sa; 9 Dairy Science and Technology Department, Faculty of Agriculture and Natural Resources, Aswan University, Aswan 81528, Egypt; osama.safwat@agr.aswu.edu.eg; 10 Department of Agricultural Microbiology, Faculty of Agriculture, Zagazig University, Zagazig 44511, Egypt; m.talaatelsadony@gmail.com; 11 Biochemistry Department, Faculty of Agriculture, Zagazig University, Zagazig 44511, Egypt

**Keywords:** purslane, polyphenol, antioxidant, serum, yogurt, physicochemical, sensorial properties

## Abstract

Purslane (*Portulaca oleracea* L.) is rich in phenolic compounds, protein, and iron. This study aims to produce functional yogurt with enhanced antioxidant, anticancer, antiviral, and antimicrobial properties by including safe purslane extract in yogurt formulation; the yogurt was preserved for 30 days at 4 °C, and then biochemical fluctuations were monitored. The purslane extract (PuE) had high phenolic compounds and flavonoids of 250 and 56 mg/mL, respectively. Therefore, PuE had considerable antioxidant activity, which scavenged 93% of DPPH˙, inhibited the viability of MCF-7, HCT, and HeLa cell lines by 84, 82, and 80%, respectively, and inhibited 82% of the interaction between the binding between Spike and ACE2 compared to a SARS-CoV-2 inhibitor test kit. PuE (20–40 µg/mL) inhibited the growth of tested pathogenic bacteria and *Candida* strains, these strains isolated from spoild yogurt and identified at gene level by PCR. Caffeic acid glucoside and catechin were the main phenolic compounds in the HPLC profile, while the main flavor compound was carvone and limonene, representing 71% of total volatile compounds (VOCs). PuE was added to rats’ diets at three levels (50, 150, and 250 µg/g) compared to butylated hydroxyanisole (BHA). The body weight of the rats fed the PuE diet (250 µg/g) increased 13% more than the control. Dietary PuE in rats’ diets lowered the levels of low-density lipoprotein (LDL) levels by 72% and increased the levels of high-density lipoprotein (HDL) by 36%. Additionally, liver parameters in rats fed PuE (150 µg/g) decreased aspartate aminotransferase (AST), alanine aminotransferase (ALT), and malondialdehyde (MDA) levels by 50, 43, and 25%, respectively, while TP, TA, and GSH were increased by 20, 50, and 40%, respectively, compared to BHA. Additionally, PuE acts as a kidney protector by lowering creatinine and urea. PuE was added to yogurt at three concentrations (50, 150, and 250 µg/g) and preserved for 30 days compared to the control. The yogurt’s pH reduced during storage while acidity, TSS, and fat content increased. Adding PuE increased the yogurt’s water-holding capacity, so syneresis decreased and viscosity increased, which was attributed to enhancing the texture properties (firmness, consistency, and adhesiveness). MDA decreased in PuE yogurt because of the antioxidant properties gained by PuE. Additionally, color parameters *L* and *b* were enhanced by PuE additions and sensorial traits, i.e., color, flavor, sugary taste, and texture were enhanced by purslane extract compared to the control yogurt. Concerning the microbial content in the yogurt, the lactic acid bacteria (LAB) count was maintained as a control. Adding PuE at concentrations of 50, 150, and 250 µg/g to the yogurt formulation can enhance the quality of yogurt.

## 1. Introduction

Milk and its products, especially yogurt, are the most consumed foods worldwide. Although milk and milk products often provide most of a consumer’s nutritional needs, other consumers prefer fortified milk and milk products with natural bioactive compounds. Various dairy products like this exist, such as whole milk, yogurt, cheese, yogurt drinks, dairy-based beverages, ice cream, milk powder, butter, and buttermilk flavor fortification ingredients. They are an excellent choice for essential nutrient fortification with natural antioxidants, i.e., flavonoids, phenolic compounds, and minerals [1,2]. The fortification of dairy products aims to overcome the lost nutrients and maintain their stability at the end of the product’s shelf life because of processing steps.

Recently, there has been an interest among health-conscious consumers worldwide in utilizing the functional and therapeutic properties of herbs and spices to maintain and improve immunity, diet, and health, especially during the COVID-19 pandemic era [3]. Phytogenic compounds as secondary metabolites from plants play an essential role in scavenging free radicals, maintaining the vitality of crucial molecules (protein, lipids, and carbohydrates), and preventing many diseases and possible food spoilage.

Synthetic antioxidants are produced by combining certain chemical compounds in a laboratory that pose health risks and cause several illnesses, including cancer, cardiovascular disease, diabetes, and others. Due to their excellent performance and wide availability [4], they are commonly employed as food additives to prevent rancidity and lipid oxidation [5]. Various studies state that the most common applications of synthetic antioxidants as food preservatives owe to their high reactivity, efficiency, and efficacy; the efficacy of synthetic antioxidants means that they have a considerable ability to scavenge free radicals, while their efficiency acts as an economical benefit, using small amounts of antioxidants. Synthetic antioxidants such as butylated hydroxytoluene (BHT) and butylated hydroxyanisole (BHA) were initially created to avoid oxidative gummification in petroleum. Nonetheless, these compounds have been utilized as antioxidants in human diets since 1954, and they are currently used in human meals to prevent oxidation. However, to protect food consumers, institutions such as the Food and Drug Administration (FDA), the European Food Safety Agency, etc., regulate their utilization. Rashmi et al. [6] noted that the amount of antioxidants authorized in food is often controlled by the lipid content of the recipient meal and is restricted to 0.02% total antioxidants. Therefore, the food sector aims to replace these additives with more cost-effective and eco-friendly natural antioxidants.

Plant antioxidants are essential in scavenging free radicals; for example, edible flowers have pharmaceutical properties and are used in some areas for culinary purposes [7]. Additionally, natural plants, i.e., pequi (*Caryocar brasiliensis*) and juçara (*Euterpe edulis*) waste extracts, Camu-Camu (*Myrciaria dubia*) peel and seed extracts, and Brazilian pepper (*Schinus terebinthifolius*), can be included in various foods, because they are rich in polyphenols [8]. Various epidemiological studies [9,10] have linked diets rich in phenolic and flavonoid compounds with considerable antioxidant activity in reducing the risk of cardiovascular disease, diabetes, cancer, and neurological disorders. In recent decades, a natural diet rich in antioxidant-active phenolic and flavonoid compounds has attracted the curiosity of food and nutrition researchers [11,12]. Moreover, several studies stimulate the cell’s creation of endogenous antioxidant molecules [13,14]. Polyphenols have been recommended as an adjuvant therapy because of their potential anti-inflammatory properties, antioxidant activity, and inhibiting enzyme systems in eicosanoid synthesis [15,16,17]. The antioxidant characteristics of plant extracts shield the product from deterioration and stop free radicals from acting on human cells, slowing aging.

Purslane (*Portulaca oleracea* L.) is an annual herbaceous plant included in the Portulaceae family and is consumed as a vegetable in Turkey; it is generated in small quantities but grows naturally in wetlands, streams, and meadows. Due to its antibacterial, antispasmodic, and diuretic characteristics, it is used in folk remedy bloody dysentery, snake and bug bites, asthma, ulcers, diarrhea, and hemorrhoids, in addition to being a tasty vegetable [18]. Fresh Purslane has been found to contain high amounts of minerals, especially potassium, vitamins E, C, and A, carotenoid derivatives, omega-3 fatty acids, glutathione, glutamic acid, aspartate, flavonoids, and phenolic compounds [19]. Therefore, *P. oleracea* has antioxidant, antibacterial, and anticancer activities [20]; it has protective effects against stomach cancer in rats, nephrotoxicity, and DNA damage [21] and nutritional benefits [22]. Purslane can safely be included in many food formulations, such as gluten-free snacks, durum wheat spaghetti, and Greek yogurt [23,24]. Purslane extract has therapeutic potential to enhance hepatic and renal functions and oxidative stress in irradiated rats [25].

Fermented foods are currently recognized as functional foods; they are novel foods formulated to contain nutrients, dietary fiber, phytochemicals, other substances, or probiotics that have possible health-enhancing or disease-preventing values. Probiotic supplements have significantly lower levels of inflammatory markers, improve gastrointestinal symptoms such as bloating and abdominal discomfort, relieve irritable bowel syndrome (IBS) symptoms, decrease lactose intolerance, and possess antibacterial and anticancer capabilities [26]. Therefore, probiotic products treat numerous health problems, including diarrhea, remove lactose intolerance symptoms, high cholesterol levels, and inflammatory diseases, and prevent cancerous changes in human cells while enhancing vitamin synthesis and immunity [27]. Yogurt is a valuable functional food containing bioactive elements, including vitamins and minerals [28]. Consequently, daily yogurt meals can become more successful at preventing illnesses caused by nutritional deficiencies and reducing the buildup of bioactive food waste as various bioactive compounds extracted from these wastes can be added to yogurt formulation. Yogurt is an excellent source of protein and calcium, but like all dairy products, it lacks Fe and vitamin C. Due to its precious nutritional content, yogurt is generally liked by customers; consequently, it serves as a carrier for various nutrients and bioactive compounds. Supplementing yogurt with prebiotics such as purslane extract in our study can increase the survival rate of probiotics and bring extra health advantages to the host [29]. Most previous studies used only purslane leaf extract to support dairy products, such as enhancing yogurt’s physicochemical, antioxidant, and sensory properties [19,30]; also, El-Sayed et al. [31] included the leaf extract as an antioxidant and antimicrobial agent to enhance Greek-style yogurt’s quality and shelf life. However, our study differs from these previous studies on many points; we used whole plant extract, not only leaves, and we comprehensively characterized the PuE extract, testing its safety and medicinal properties, then included it in a new yogurt drink sweetneed by the tested extract.

Therefore, this study aimed to characterize purslane extract as an in vitro antioxidant, anticancer, antiviral, and antimicrobial agent and evaluate its HPLC phenolic compound profile and GC-MS volatile compounds. The safety of *P. oleracea* extract (PuE) was compared to BHA in rats’ diets. PuE was added to the yogurt formulation, and the fluctuation in physicochemical, sensory, color, and microbial quality were monitored during the storage period of four weeks.

## 2. Results and Discussion

### 2.1. Proximate Analysis of Purslane Plant

The proximate composition of the whole purslane plant, including moisture, ash, crude fat, crude fiber, carbohydrates, crude protein, and minerals content, was determined in this study. Table 1 shows that purslane is rich in carbohydrates, protein, and fibers, with 44.3, 18.2, and 21.6%, respectively. Mineral analysis showed that the purslane plant is rich in potassium (5033 ppm), magnesium, and calcium (695 and 671 ppm). Our results are similar to Obied et al. [32], who found that purslane had a high protein and crude fiber content and lower fat. Additionally, our results correlate with Uddin et al. [33]; they found that purslane leaves had considerable amounts of K, Mg, and Ca, similar to ours. On the other hand, the mineral content in our results exceeds those of de Souza et al. [34]. Purslane has lower protein and ash content, as stated by Petropoulos et al. [35]. The differences in proximate composition between different studies depend on the plant part, extraction method, solvent, and other conditions.

Table 1 shows that the content of phenolic compounds and flavonoids in purslane extract was 250 and 56 mg/g, respectively. The phenolic content in our results increased four-fold compared to the results of Fernández-Poyatos et al. [36] concerning the purslane leaf extract. Additionally, Karoune et al. [37] stated that the methanolic extract of purslane is rich in polyphenols, but their content is lower than the obtained results in this study. The variation in polyphenols depends on several factors, i.e., environmental conditions and growing soil [38].

### 2.2. Detection of Phenolic Compounds in Purslane Extract by LC/MS

The phenolic compounds in PuE were described by mass spectrometry employing both positive and negative ion modes; the most detected compounds were discovered using the negative method (Table 2). The LC/MS spectra distinguished 31 phenolic compounds; phenolic acids were the main compounds in the LC/MS profile, accounting for 51%, flavonoids 33%, tannins 11%, and phenolic derivatives 5%.

Caffeic acid glucoside is the main phenolic acid (30.96 mg/100 g). It was detected at deprotonated molecular ions at 339 *m*/*z* and fragment ions at 133 and 177 *m*/*z*. This fragmentation pattern has been documented in the library for Caffeic acid glucoside and Caffeoyl glucose. Consequently, quadrupole time-of-flight (Q-TOF) was used to determine the exact mass, comparing the fragmentation pattern with the METLIN database. So, Caffeic acid glucoside was detected at retention time (RT, 10.55). Similarly, coumaric acid glucoside and neochlorogenic acid were found at RT 11.30 and 16.30. Concerning flavonoids, catechin was the main compound (26.65 mg/100 g) detected at molecular ion 287 *m*/*z* and confirmed at fragment ion (243 *m*/*z*) by Q-TOF, followed by gallocatechin at 302 *m*/*z*. Furthermore, Galloyl glucose was the prominently detected tannin with 11.2 mg/100 g, seen at molecular ion 329 *m*/*z*, followed by caffeoyl glucose at 338 *m*/*z*. On the other hand, PuE showed its richness in organic acid at molecular ion 190 and fragmented ions at 110 and 111 *m*/*z*, which correlated to quinic and citric acids with 1100 mg/100 g. Purslane is rich in flavonoids and phenolic acids, i.e., caffeic acid, p-coumaric acid, ferulic acid, apigenin, quercetin, kaempferol, luteolin, isorhamnetin, kaempferol-3-O-glucoside, and rutin [39]; it was found that quercetin content was in the range of 6–16 mg/kg, rutin was 4–6 mg/kg, and kaempferol was 2–4 mg/kg; however, our results defeated these content results. On the other hand, our results are in agreement with Fernández-Poyatos et al. [40], who detected 24 phenolic compounds, including phenolic acids, organic acids, and flavonoids; the main compounds in purslane extract were isocitric and citric acids, with concentrations of 550 and 600 mg/100 g of the purslane extract. The compounds were detected by HPLC coupled with a diode array and mass spectrum detectors.

### 2.3. Volatile Compounds in Purslane Extract

Purslane aqueous extract is rich in volatile compounds related to alcohols, hydrocarbons, aldehydes, ketones, nitrogen compounds, mono, and sesquiterpenes (Table 3). Based on these findings, terpenoids represent 81.2% of total VOCs, followed by oxygenated compounds (11%) and hydrocarbons (6.5%) in the PuE flavor profile. The main volatile compounds in PuE were carvone and limonene, which represented 71% of total VOCs, followed by 2,6-dimethylcyclohexanol (5.2%), cuminal, and β-caryophyllene (2.2%); other GC profiles were found at lower contents. Fukalova et al. [41] found that the VOCs profile of purslane was rich in monoterpenoids (48–55%) and fatty alcohols (16–54%). Additionally, Almashad et al. [42] found that purslane leaf extract contained high levels of (*E*)-2-hexenal (15.64%), (*E*)-2-nonenol (12.03%), hexanal (10.92%), and ethyl linoleate (8.02%). In contrast, the main VOCs recovered by water distillation (WD) were (*E*)-2-hexenal (12.46%), (*E*)-2-nonenol (9.52%), hexanal (9.32%), and menthol (8.04%). Terpenoid compounds, mono, and sesquiterpenes, belong to the phenolic compounds enclosed in several plants and are considered secondary metabolites that play a significant role in antioxidant activities [43]. Additionally, oxygenated and nitrogen compounds are known for their scavenging activity, so *P. oleracea* extract might be a source of phytochemicals and antioxidants.

### 2.4. Biological Activity of PuE

#### 2.4.1. Scavenging Ability

**Figure 1:**
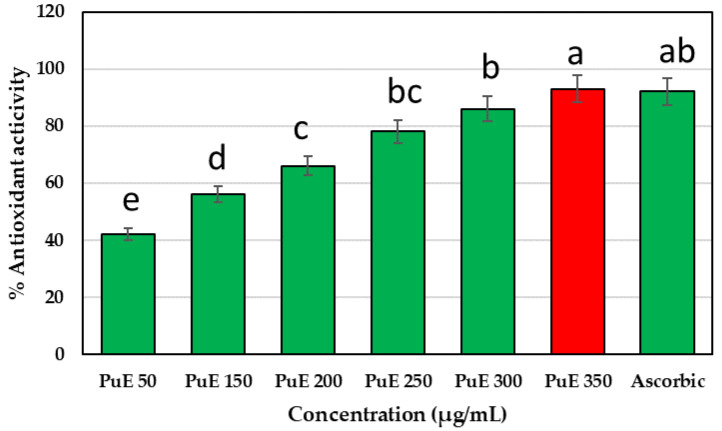
% Scavenging activity of *P. oleracea* extract against DPPH free radicals. Ascorbic acid concentration (350 µg/mL). Lowercase letters (a–e) above columns indicate significant differences between the antioxidant activity of purslane extract and ascorbic acid against DPPH free radicals using the LSD test at *p* < 0.05.

The high radical scavenging activity of PuE is attributed to the high phenolic, flavonoid, and volatile compounds content in purslane [11]. The antioxidant activity of PuE increased in concentration dependant manner, where it considerably scavenged 93% of DPPH free radicals compared to ascorbic acid (Figure 1). The lowest concentration of PuE that scavenged 50% of free radicals (SC_50_) was 150 µg/mL. Uddin et al. [44] determined that *P. oleracea* extracts scavenged 76.71% of the DPPH radical. In our study, PuE water extract scavenged the DPPH radical by 93%, respectively, and it was observed that our results remained at a higher rate compared to the study of Uddin et al. [44]. Concerning SC_50_, our results excelled those of Wang et al. [45], who found that purslane extract exhibited free radical scavenging activities, with SC_50_ of 5.11 mg/mL for DPPH radicals, which was lower than our study. In the current study, polyphenols, flavonoids, volatile compounds, and organic acids such as chlorogenic acid, caffeic acid glu, catechin, and quercetin rham may be responsible for the antioxidant activity in purslane leaf extracts, and the differences in phenolic compounds content are attributable to the variation in our results compared to those of previous studies.

#### 2.4.2. Cytotoxic Effect

**Figure 2:**
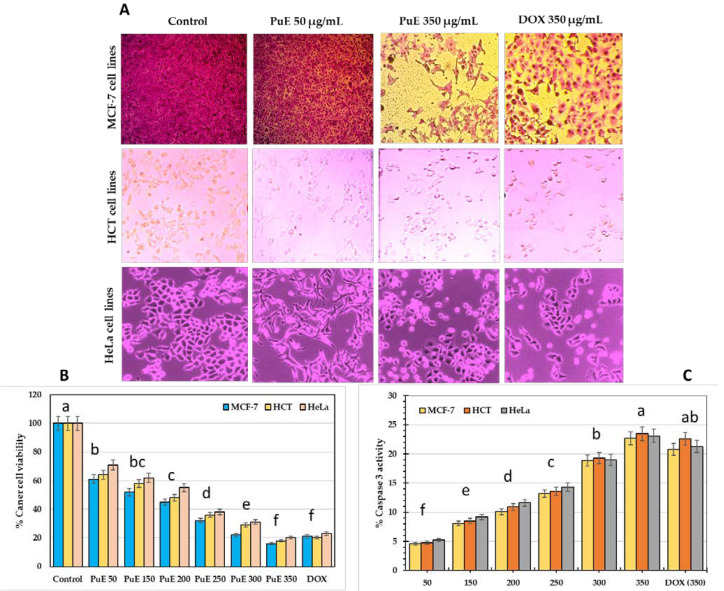
(**A**) Microscope image of the influence of *P. oleracea* extract concentrations on the viability of breast cancer cell lines (MCF-7), colon cancer cell lines (HCT), and cervical cancer cell lines (HeLa). (**B**) Histogram of % viability of cancer cell lines affected by PuE with different concentrations comparing doxorubicin (DOX, 350 µg/mL); (**C**) The activity of apoptotic marker, caspase-3, on cancer cell lines as affected by PuE concentrations. Lowercase letters (a–f) above columns indicate significant differences between the cytotoxicity and apoptotic effect of purslane extract and DOX against cancerous cell lines using the LSD test at *p* < 0.05.

Figure 2 shows that PuE has significant anticancer activity against the breast cancer cell line (MCF-7), colon cancer cell line (HCT), and cervical cancer cell line (HeLa) compared to the anticancer medicine doxorubicin (DOX). The results indicate that the viability of tested cancerous cells decreased while purslane extract increased (*p* ≤ 0.05); PuE (350 µg/mL) hindered the viability of MCF-7, HCT, and HeLa cell lines by 84, 82, and 80%, respectively, compared to DOX with 80, 79, and 77% (Figure 2B). These inhibition percentages are apparent in the microscopic images (Figure 2A), demonstrating that the cytotoxicity effect against cancer cell lines is higher in PuE than DOX at a concentration of 350 µg/mL. Our results indicate that PuE vindicates the oxidative stress on human cells, correlating with Farshori et al. [46], who found that exposing liver cancer cell lines (HepG2) to purslane extract diminished their normal morphology and adhesion. HepG2 cancer cell lines treated with 50 µg/mL and greater concentrations of purslane extract lost their standard shape, shrank in size, and took on a rounded appearance. The preliminary screening of the anticancer activity of *P. oleracea* extracts against HepG2 cells can be exploited to build a possible anticancer treatment drug. Additionally, Keser et al. [47] found that purslane extract exhibited potent anticancer activity against MCF-7, HCT-116, and prostate cancer cell lines (PC-3).

By analogy with the use of active substances from plants with anticancer properties, an essential bioactive flavonoid of the Epimedium plant, Icariin, plays a decisive role by increasing cytochrome c secretion, Bax/Bcl2 ratio, poly (ADP-ribose) polymerase, and caspase stimulations. Surprisingly, it can induce apoptosis, reduce viability, and inhibit the proliferation of cancer cells [48]; also, *Ferula gummosa* gum, as a capping agent in nanoparticles, has a significant cytotoxic effect on breast cancer cells and little toxic activity on normal cells [49]. Additionally, resveratrol is an active polyphenol that plays a considerable role in fabricating nanoceria and has significant cellular toxicity properties against HepG2 [50].

Figure 2C shows that the activity of caspase-3 increased as an indicator of cancerous cells’ death in a concentration-dependant manner, showing high activity when breast, colon, and cervical cancerous cell lines were treated with PuE extract (350 µg/mL), where activity was estimated as 22.69, 23.5, and 23.1%, respectively, compared to DOX (20.8, 22.6, and 21.3%, respectively). The results indicated that HeLa cells were more vulnerable to apoptosis by PuE concentration than other cancerous cells following the Nichani et al. [51] study. Caspase-3 is essential for normal brain development and other apoptotic conditions in a tissue-, cell-, or death-stimulus-specific manner. It is also necessary for apoptotic chromatin condensation and DNA fragmentation in every examined cell type. Caspase-3 is essential for the breakdown of cells and the production of apoptotic bodies and may activate before or during death cell [52].

#### 2.4.3. Antimicrobial and Antiviral Activity

The purslane extract showed considerable antibacterial efficacy against the tested pathogenic bacteria and *Candida* (Table 4). The inhibition zone diameters (IZDs) of PuE increased in a concentration-dependent manner, in the range of 1.0–4.2 cm against the tested bacteria and 1.2–3.3 cm against tested *Candida,* with a relative increase of 6–8% over bacterial or fungal antibiotics. *Staphylococcus aureus* (SA) was the most susceptible G+ bacteria to PuE 350 µg/mL (4.2 cm), while *Klebsiella pneumonia* (KP) was the most resistant G− bacteria (2.8 cm). *Candida gelberta* (CG) was the most resistant to PuE 350 µg/mL in the recorded inhibition zone (2.9 cm), followed by *Candida albicans* (CA) with 3.1 cm.

Our findings agree with Kesar et al. [47], who discovered that the aqueous and methanolic extract of *P. oleracea* exhibits antimicrobial activity against pathogenic bacteria and *Candida*. On the other hand, *P. oleracea* ethanolic extract recorded lower IZDs than our study (1.0 cm) against *B. subtilis;* in addition, methanolic extract of purslane recorded 1.1, 0.9, 1.0, and 1.1 cm, respectively, against *E. coli*, *L. monocytogenes*, *K. pneumoniae,* and *B. subtilis* compared to the standard antibiotic.

**Figure 3:**
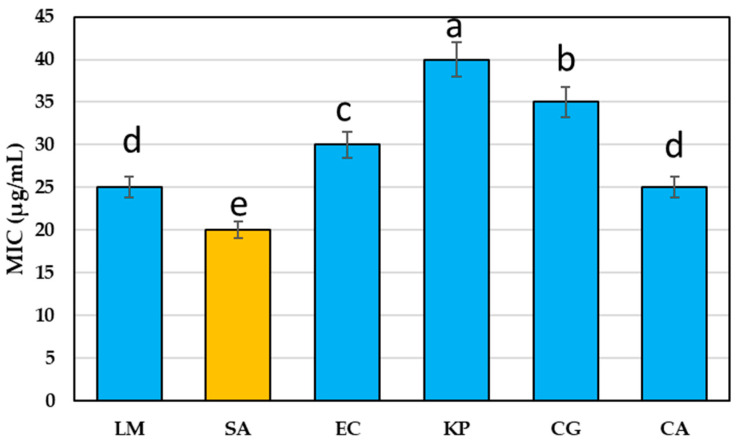
The minimum inhibitory concentration (MIC) of *P. oleracea* extract (PuE) against tested bacteria and *Candida* strains. Lowercase letters (a–e) above columns indicate significant differences between MIC of *P. oleracea* extract (PuE) against tested microbes using the LSD test at *p* < 0.05.

The lowest concentration of purslane extract against the tested pathogenic bacteria and fungi was 20 to 40 µg/mL (Figure 3). The minimum inhibitory concentration (MIC) against SA was the lowest (20 µg/mL), while it was the highest against KP (40 µg/mL). Our results showed more potent antimicrobial activity than the study of Tleubayeva et al. [53], who found that the MIC of CO_2_-purslane was in the range of 250–500 µg/mL against *E. coli*, *S. aureus, B. subtitles*, and *C. albicans.*

Also, PuE extract showed an antiviral activity on the binding between Spike and ACE2 compared to a SARS-CoV-2 inhibitor test kit. At 50-350 µg/mL doses, PuE extract inhibited the interaction between Spike and ACE2 by 82%. This impact was a dose-dependent compared to the positive control, AC384, a monoclonal antibody that prevented the binding between Spike and ACE2 by identifying ACE2 itself and inhibiting 75% of the interaction. 

### 2.5. Safety Experiment

#### 2.5.1. Body Weight Gain and Final Weight

PuE treatments significantly (*p* < 0.05) influenced final weight (FW) and body weight gain (BWG) (Supplementary Table S1); the rats fed a diet containing PuE (250) had the best FW (315 g) and BWG (68.2 g), respectively, compared to the BHA group, which had FW of 298.3 g and BWG of 51.8 g. The enhanced growth parameters of rats receiving dietary PuE, i.e., FW and BWG, are attributed to active components such as flavonoids, polyphenols, and alkaloids of *P. oleracea* extract. Polyphenols have been reported to stabilize the antioxidant activity of membranes by inhibiting the generation of reactive oxygen species (ROS) and maintaining the cell membrane structural integrity [54]. Our results agree with Seif et al. [54], who found that including purslane extract in rats’ diet at 2000 mg/kg significantly enhanced the BWG of 75 g compared to 68 g in control samples. Additionally, Wang et al. [55] found that adding purslane to a broiler diet enhances growth performance. From the results, PuE is safer and more efficient than synthetic antioxidant BHA in improving growth performance.

#### 2.5.2. Serum Biochemical Parameters

Table 5 shows that all biochemical markers assessed in BHA diet rats were higher (*p* < 0.05) than in the other groups. The serum AST, ALT, creatinine, and urea concentrations were significantly lower in rats fed PuE for four weeks; furthermore, the total protein, albumin, and GSH levels in the serum of PuE-treated rats increased compared to the control and BHA groups. The high content of phenolic and bioactive components in purslane extract preserves the plasma membrane in hepatocytes. It protects it from rupturing and exiting the cytosol loaded within these enzymes. Additionally, the phenolic content decreased aminotransferase enzyme levels and hepatocytes’ restoration of some essential functionalities [56].

Seif et al. [54] observed that purslane reduced liver damage caused by H_2_O_2_, as evidenced by a substantial reduction in ALT and AST levels in serum blood and liver homogenates and increased total protein and albumin levels. Additionally, Mousa et al. [57] found that supplementation with fresh purslane, particularly at a concentration of 75%, protects against the harmful effects of a high-fat diet at both the cellular and organ levels. In addition, the evaluated liver functions, thyroid hormones, and cholesterol profile improved. Fresh purslane can act as a hypolipidemic drug, suggesting that it can be used to combat the negative consequences of obesity.

MDA, the lipid oxidation indicator increased in the BHA group, was 67.9 nmol/mL compared to the control of 46.36 nmol/mL. There was also a significant decrease in rats fed PuE (150 µg/g) with 51.22 μmol/L. MDA production and accumulation can result in oxidative processes and inhibitory and cytotoxicity effects, damaging cell membranes and changing their structure and function [58,59,60]. Seif et al. [54] found normal MDA in rats receiving a diet supplemented with purslane extract, confirming the antioxidant activity of PuE.

Additionally, BHA exhibited a substantial rise in kidney markers (urea and creatinine) (*p*< 0.05), as seen in Table 5, compared to the control and PuE groups. The PuE rats had a substantial drop in urea and creatinine levels compared to the BHA group. Creatinine and urea levels were lowered in the rat group fed PuE (150 µg/g) compared to BHA and control rats. Seif et al. [54] discovered that supplementing the diet with purslane extract reduced urea, creatinine, and malondialdehyde compared to a positive control group, verifying our findings. Because PuE is a relatively high natural antioxidant substance, rats fed PuE (150 µg/g) showed a considerable decrease in kidney function markers (creatinine and urea) compared to BHA rats. These natural antioxidants can decrease urea and creatinine in blood serum through clear uricosuric potential or improve renal blood flow.

Table 5 shows that adding dietary PuE at three levels (50, 150, and 250 µg/g) significantly reduced the lipid profile, triglyceride, LDL, and cholesterol levels and enhanced HDL levels in a concentration-dependent manner. The rats fed a diet supplemented with PuE of 150 µg/g had the lowest cholesterol levels (72.6 mg/dL) compared to the BHA rats with the highest levels (135.2 mg/dL). Additionally, the rats fed a diet supplemented with PuE (150 µg/g) had lower levels of triglycerides and LDL (79.6 and 23.5 mg/dL, respectively) compared to the BHA group that had the highest levels (119.2 and 85.4 mg/dl, respectively). The control group had the lowest HDL content of 25.40 mg/dL compared to the other groups. However, the rats treated with PuE had increased HDL content of 37.3 mg/dL.

High concentrations of synthetic antioxidant BHA may cause health problems because of safety concerns, and, as a result, interest in natural antioxidants has intensified [60,61]. The study findings revealed that purslane extract had a beneficial effect on the lipid profile because of the phenolic compounds in purslane leaves, which possess potent antioxidants [54]. Seif et al. [54] found that the TG and TC of rats fed a diet supplemented with PuE 2000 mg/kg were 52 and 177 mg/dl, respectively, with no significant differences compared to the control. We conclude from the results presented in Table 5 that PuE is safer than BHA as an antioxidant agent.

#### 2.5.3. Protective Effect of PuE against Lipid Oxidation in Rat Brain

**Figure 4:**
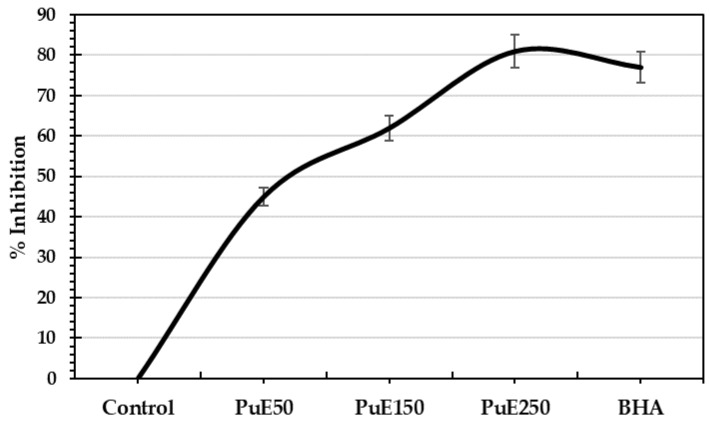
The inhibition effect of *P. oleracea* extract on Fe^2+^-induced brain lipid peroxidation in rats.

The inhibition percentage of purslane extracts on Fe^2+^-induced brain lipid peroxidation in rats compared to BHA is reported in Figure 4. The PuE extract significantly decreases brain lipid peroxidation in a concentration-dependent manner (Figure 4). The maximum % inhibition was 81% in PuE (250 µg/g) compared to 77% in BHA. Due to its antioxidant activity, the phenolic compounds and vitamin C in the purslane extracts could inhibit Fe^2+^-induced brain lipid peroxidation in rats. High concentrations of bioactive compounds, i.e., omega-3, ascorbic acid, phenolic compounds, and fatty acids in PuE, provide it with protective effects against the degeneration of dopaminergic neurons [62] and oxidative stress by decreasing glutathione levels [63].

### 2.6. The Experiment of Yogurt Supplemented with P. oleracea Extract at Different Concentrations (50, 150, and 250 µg/g)

#### 2.6.1. Proximate Composition of Yogurt Supplemented with *P. oleracea* Extract

As stated in Table 1, purslane is rich in carbohydrates, protein, and fibers, with 44.3, 18.2, and 21.6%, respectively; therefore, these components significantly increased in yogurt samples supplemented with PuE, where the fiber content increased by 40%, protein by 25%, and polysaccharides by 45% compared to the control. The increase in polysaccharides considerably enhanced the taste of the yogurt samples. Our results are in agreement with those of Valencia-Avilés et al. [64].

#### 2.6.2. Physiochemical Parameters

Table 6 showed that supplementing yogurt with PuE at three concentrations (50, 150, and 250 µg/g) significantly enhanced the physiochemical properties compared to the control during monthly storage. The pH value in the control yogurt decreased to 4.11 after 30-day storage, but adding PuE at 150 and 250 µg/g panned this decrease and kept pH at approximately 4.30 and 4.23, respectively. The results showed that pH decreased by 10% in the control compared to 5–7% in the PuE-yogurt samples (50, 150, and 250 µg/g). The decrease in pH was followed by an increase in acidity by 13% and 9% in the control and PuE-yogurt drinks. Fat content increased by 59% in the yogurt sample supplemented with PuE (150 µg/g) compared to the control yogurt (Y control). The gradient concentration of PuE had no effect on the time necessary for yogurt mixtures to achieve a pH of 4.4, and the starting culture populated and generated lactic acid, which reduced pH and increased acidity; we noticed that PuE 150 µg/g had the best performance. The results also showed that TSS increased by 12% compared to the control.

The increase is attributable to the evaporation of yogurt whey during cold storage [65] or the conversion of rich polysaccharides into simple sugar [66]. Total sugars decreased due to the breakdown of the total sugars by acid-producing bacteria, increasing TSS [67]. Generally, in any preservation period of any food product, some properties increase, and others decrease, i.e., pH is decreased while acidity, fat, and total soluble solids (TSS) are increased [68].

The addition of fiber-enriched materials such as PuE (250 µg/g) decreased syneresis by 20% compared to the control because of the high water-holding capacity (WHC) of purslane (9.5 mL/g); conversely, viscosity increased in PuE-yogurt by 122% compared to the control. Therefore, the WHC of PuE-yogurt increased by 16–30% compared to the control yogurt depending on the additional level of PuE.

The sodium caseinate and maltodextrins molecules in PuE-yogurt coated the purslane, indicating the probability of binding these compounds with water, increasing WHC and its ability to retain moisture, increasing TSS, decreasing syneresis, and increasing viscosity. Numerous studies have demonstrated that the rheological properties of yogurt differ by fiber type and source [69]. The ability of fibers to increase water holding capacity (WHC), stabilize high-fat yogurt, improve viscosity qualities, and create gels permits the production of fiber-enriched yogurt with improved texture and less syneresis [70]. Additionally, regarding the moisture changes caused by PuE concentrations, a substantial increase in fat content was detected between untreated and treated yogurts after four weeks of cold preservation (*p* < 0.05) [65]. Our results agree with Osman et al. [71], who included purslane extract at four levels 0.0, 0.1, 0.2, 0.3, and 0.4% in ice cream and found a decrease in pH and total carbohydrates. Additionally, Salahi et al. [30] found that supplementation of yogurt with purslane leaf extract and preservation for 21 days at 4 °C significantly affected the physicochemical and sensory properties (*p*  <  0.05). It was also shown that the sample containing 2% extract had lower acidity than others during storage. Furthermore, adding extracts at all levels reduced syneresis and the lowest amount was found in the sample containing 1.5% extract. Furthermore, increasing the extract ratio in the samples increased viscosity significantly.

#### 2.6.3. Antioxidant Content in Yogurt Samples Supplemented with Purslane Extract

**Figure 5:**
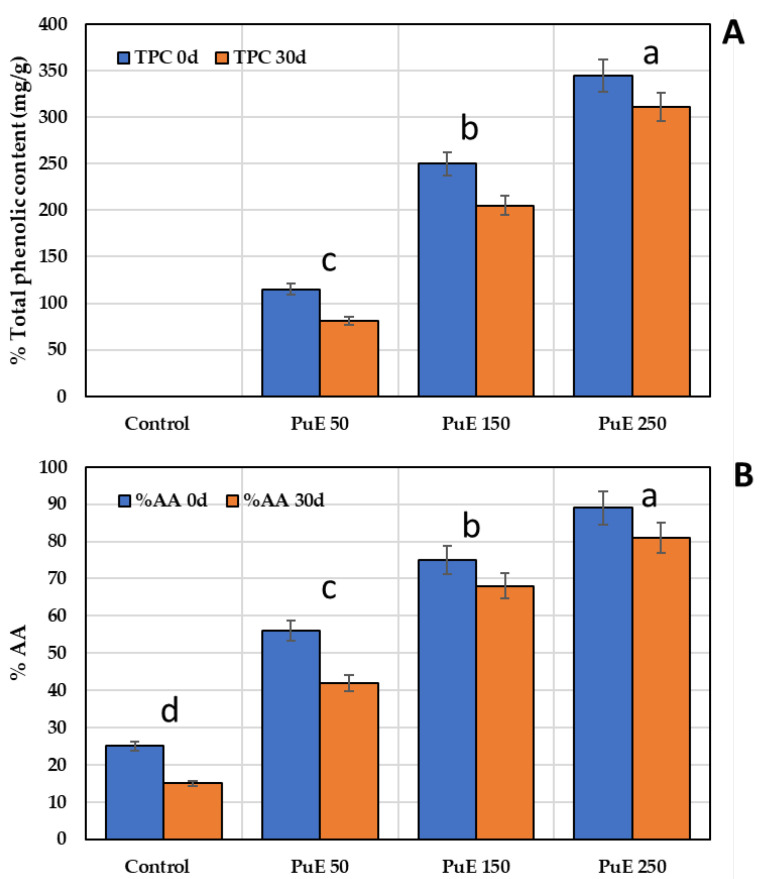
(**A**) Total phenolic content of yogurt after adding different concentrations of purslane extract. (**B**) Antioxidant activity of yogurt samples supplemented with purslane extract. Lowercase letters (a–d) indicate the significant differences between the polyphenols and antioxidant content of PuE yogurt samples compared to the control using the LSD test at *p* < 0.05.

Figure 5A shows that adding PuE to the yogurt formulation significantly (*p* < 0.05) increased the phenolic content in the yogurt samples compared to the control. The increase was estimated to be two-fold higher the control yogurt at the beginning and end of storage. This is the same trend observed in the TFC of yogurt because flavonoids are a division of phenolic compounds. Figure 5A shows that the phenolic content in the yogurt samples decreased with an increase in the storage period, but the increase in supplemented samples was slower than the control. The phenolic compounds in PuE bind with protein in yogurt, increasing the antioxidant potential of yogurt [72]; this product can be proposed as a novel functional food that can fulfill customers’ nutritional requirements.

Figure 5B shows the antioxidant status of yogurt samples before and after adding PuE at three concentrations (50, 150, and 250 µg/g) during the storage period of 30 days. The results show that DPPH-scavenging activity increased with PuE addition levels, where the antioxidant activity in PuE 250-yogurt increased 2.5-fold over the control on the first day of storage; % AA continued to increase by 4.4-fold at the end of storage. Our results agree with Guemidi et al. [73], who found a significant percentage increase in the antioxidant activity of yogurt, 40%, compared to the control yogurt when *Mentha piperita* extract was added to the formulation. Cho et al. [74] reported a reduction in the scavenging activities of DPPH radicals during storage, which may be because a complex was formed between the polyphenols in the extract and the yogurt proteins, thereby reducing the restoration of the polyphenols and their antioxidant effect [72].

#### 2.6.4. Lipid Oxidation of Yogurt Samples during the Storage Period

Table 7 shows that the TBA test includes the formulation of the interaction between MDA “produced by lipid oxidation” and TBA reagent, producing pink color. The oxidation of polyunsaturated fatty acids in yogurt produces a pink color absorbed at 532 nm [75]. The ability of PuE to mitigate oxidative stress can be measured by MDA content (Table 7). The results indicate that MDA content increases in correlation with the storage period; however, PuE-supplemented yogurt witnessed a slow increase in MDA. The % MDA in the control yogurt was 92%, which decreased with gradient supplementation of PuE in yogurt to 58% in PuE (50 µg/g), 40% in PuE (150 µg/g), and 31% in PuE (250 µg/g). Therefore, adding PuE (250 µg/g) to yogurt decreases lipid oxidation by 69% after 30 days of storage

Adding PuE to yogurt is expected to reduce oxidation and aldehyde formation compared to the control, with considerable differences (*p* < 0.05) based on PuE concentrations. The high PuE concentration generally had lower TBA absorbance compared to the control sample. Purslane extract inhibited the oxidation of rabbit patties’ lipids and proteins during cold storage [45].

#### 2.6.5. Color Properties

Color properties were enhanced by adding PuE, especially at a concentration of 150 µg/g; lightness (*L*) was improved compared to the control; blueness was enhanced by 33% compared to the untreated yogurt. After 30 days of cold storage, the color properties defatted, where lightness decreased by 4% with no significant differences from the control. During the preservation period, the color change in PuE-yogurt was from 1.45 to 1.17, compared to 1.87 for the control yogurt (Y control), indicating the antioxidant properties of PuE in maintaining color from oxidation (Supplementary Table S2).

#### 2.6.6. Texture Properties

The texture properties (firmness, consistency, and adhesiveness) increased with the increase in storage periods and gradual PuE levels (Supplementary Figure S1). The best texture was observed in PuE-yogurt supplemented with 150 µg/g compared to the control, with relative increases of 17, 24, and 26% for firmness, consistency, and adhesiveness, respectively. Conversely, El-Syed et al. [31] stated that the addition of purslane leaf extract reduces the texture properties because the leaves are rich in polysaccharides; however, our PuE is rich in fiber which binds with sodium caseinate and maltodextrins molecules, and consequently with water, increasing the WHC of yogurt and its ability to retain moisture, increasing TSS and texture properties.

#### 2.6.7. Sensorial Properties of Yogurt Drink Samples

The sensory evaluation of yogurt enriched with PuE at three levels (50, 150, and 250 µg/g) during 30 days of cold storage at (4 °C) is presented in Table 8. Compared to the control, PuE yogurt remarkably kept the yogurt’s color. The results demonstrated that adding PuE at 50, 150, and 250 µg/g to fermented milk formulation produced the best color and flavor. However, some studies stated that the addition of 2% mint extract does not alter the natural white color of the control yogurt, whereas increasing levels to 4% and 6% resulted in more intense and less pleasant colors, likely due to the pigments contained in the extract that are responsible for characteristic plant dyes [76]. There were no significant differences between 150 and 250 µg/g concentrations; therefore, from an economic point of view, we recommended using 150 µg/g as we used fewer material resources and obtained the best results. Other sensory aspects were significantly different from the control. Yogurt supplemented with PuE 250 µg/g showed the lowest sensory characteristics: color, shape, flavor, texture, and overall acceptability. The overall acceptability of PuE-yogurt (50 and 150 µg/g) was 8.1 and 8.3, respectively, and declined with gradient increments and storage duration, reaching 7.9 in PuE-yogurt (250 µg/g) after 30 days of storage with no significant differences when increasing PuE concentration. Our results may agree with El-Sayed et al. [31], who fortified Geek-style yogurt with lyophilized purslane leaf extract at two levels, 2.2 and 4.4 g/L. The highest concentration of lyophilized purslane extract, 4.4 g/L, showed the lowest sensory acceptability compared with the control and lyophilized purslane extract (2.2 g/L), which was the most preferred choice concerning sensory acceptability.

All yogurt samples earned the highest sensory scores at the beginning of preservation because of their improved flavor intensity and consistency. However, in the end, the microbial load increased as the acidity of the yogurt increased, and the sensory scores progressively dropped. After 21 days of storage, the overall quality of yogurts rose before declining. Thus, it can be linked to the generation of acidity. After storage, the sensory properties of yogurt supplemented with varying PuE levels were generally stable. These results may be used to make yogurt with improved antibacterial, antioxidant, and anticancer qualities without affecting the sensory traits of yogurt, boosting the flavor quality of the yogurt.

#### 2.6.8. Lactic Acid Bacteria (*Starter bacteria*) Count

**Figure 6:**
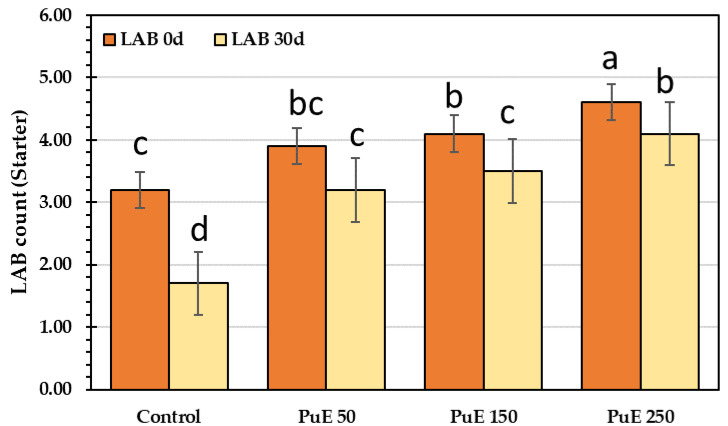
The total lactic acid (starter) bacterial count of control and *Portulaca oleracea* extract (PuE) yogurt samples during cold storage (0–30) days. Lowercase letters (a–d) above columns indicate significant differences in LAB count in yogurt affected by *P. oleracea* extract concentrations during cold storage of 30 d at *p* ≤ 0.05 using the LSD test. LAB, lactic acid bacteria count.

Figure 6 illustrates that all PuE-supplemented samples contained enough quantities of viable lactic acid bacteria for up to 30 days of storage; therefore, PuE-yoghurt samples are acceptable for consumption for up three weeks. The lactic acid bacteria (LAB) count reduced during storage, where the LAB count declined by 40% in the control sample but increased by 82% in the PuE 250 µg/g compared to the control sample. The count of probiotic bacteria (*B. lactis, L. acidophilus,* and *S. thermophilus*) in the yogurt after 30 days of cold storage at 4 °C may be connected to the low pH caused by increased acidity. There is mutual stimulation between the aromatic compounds’ development of acidity production and the yogurt starter. After fermentation, organic acids (such as lactic and acetic acid) accumulate, and LAB increases. Hu et al. [77] stated that organic acids are the predominant antibacterial agents. This study indicated that adding PuE to yogurt samples promotes the growth of probiotics (Figure 6).

## 3. Materials and Methods

### 3.1. Experimental Materials

The purslane plants were collected in July 2022 from a privte farm. The reagents, Folin reagent, gallic acid, sodium carbonate, AlCl_3_, methanol, acetonitrile, anhydrous MgSO_4_, DPPH, acetic acid, HCl, thiobarbituric acid, NaOH, phenolphthalein, and sulphuric acid, were purchased from Sigma. Muller Hinton medium (Oxiod, Basingstoke, UK). All chemicals and kits were of analytical grade.

### 3.2. Chemical Composition of Purslane

The whole purslane plant was dried in a Thermolyne oven (USA) at 50 °C and then powdered. The moisture, ash, crude protein, and fat levels were determined by procedures 926.08, 942.05, 991.20, and 2000–2018, respectively, according to AOAC [78]. The dietary fiber content was determined using the enzymatic approach developed by Moczkowska et al. [79]. The difference measured the carbohydrates content:

(1) $$\%\;\mathrm{Carbohydrates}=100-\left(\%\;\mathrm{protein}+\mathrm{fat}+\mathrm{ash}+\mathrm{mositure}\right).\;$$

### 3.3. Preparation of Purslane Extract (PuE)

Collected fresh purslane plants were extracted using solid–liquid extraction [80]. The plant was washed with distilled water, dried (temperature, 25 °C, relative humidity, 28%), cut into pieces, and then dried in a Thermolyne oven (USA) at 50 °C for two days until the moisture reached 10%. The dried plants were ground to a fine powder using a mill (Moluniex DPA144, Paris, France) and then sieved to obtain a particle size of 40 mm; 100 g of dried plant material were obtained from 1 kg of fresh plant. The fine powder (10 g) was stirred using a magnetic stirrer (150 rpm, Bexco, Haryana, India) in deionized water (100 mL) as a solvent for 24 h at 25 °C. The extract was filtrated through filter paper (Whatman no. 1) and re-homogenized in water. The aqueous extract was evaporated at 50 °C using a rotary evaporator under a vacuum (Heidolph Rotary Evaporator, Schwabach, Germany). The final concentration was 100 mg/mL. Different concentrations (50, 150, 200, 250, 300, and 350 µg/mL) were prepared from the intact concentration. Part of the concentrated extract was frozen at −60 °C in a lyophilization flask. The lyophilizer was switched on until the temperature was −60 °C under pressure. The process was completed using a Heto PowerDry LL3000 Freeze Dryer (Thermo Fisher Scientific, Waltham, MA, USA) to obtain the powdered PuE.

### 3.4. Polyphenolic Content in Purslane Extract

#### 3.4.1. Total Phenolics (TPs) and Flavonoids (TFs)

TPs and TFs in PuE were measured by a Biotek microplate reader (BioTek Elx808, Winooski, VT, USA) at wavelengths of 750 and 430 nm, respectively [81,82]. TPs were estimated with the Folin method, as follows, 100 µL of PuE were added to the respective wells, then 50 µL of sodium carbonate (7.5%) and 50 µL of diluted Folin reagent were added to each well and incubated for 30 min at 55 °C. The deep blue color was measured at 750 nm, and the total phenolic compounds’ content was expressed as a mg equivalent of gallic acid (GAE) per gram of extract using the gallic acid standard curve.

The TFs were evaluated by the AlCl_3_ method as follows, 100 µL of PuE were added to the respective wells, then 50 µL of ethanolic AlCl_3_ were added and kept in the dark for 30 minutes. The developed yellow color was measured at 430 nm, and TFs in PuE were expressed as a mg quercetin equivalent per extract using a standard quercetin curve.

#### 3.4.2. Phenolic Compounds Profile by LC-MS/MS

Five grams of powdered PuE were homogenized in 100 mL methanol (HPLC grade 99.8%) and centrifuged (SIGMA 3-30K, GmbH, Berlin, Germany) at 10,000 rpm for 10 min. The supernatant was filtered through a 0.2 µm Millipore membrane filter, and approximately 1–3 mL of the filtrate were collected and kept for HPLC application. The phenolic content was analyzed by HPLC (LC-10AS, Shimazu, Japan) equipped with an autosampler, solvent degasser, and quaternary HP pump column (C18, Gemini, 4.60 mm, 5 μm, 35 °C). The mobile phase flow rate of 1 mL/min was determined with a triple-quadruple spectrometer (LCMS 8040, Shimazu, Japan) connected to an electrospray ionization (ESI) source. The gradient solvent system was water (A) and acetonitrile (B) 5–60% in formic acid. Isocratic elution was 95% A and 5% B for the first 5 min, followed by a linear gradient to 50% A and 50% B between 5 and 55 min. It was also 50% A and 50% B between 55 and 65 min, and the liner gradient returned to 95% A and 5% B between 65 and 67 min. The samples were automatically injected using the autosampler SIL-40Cxs (Shimadzu, Kyoto, Japan). The data were managed using LC solution software (Shimadzu), and MS functioned in negative mode. In total, 35% of the collision energy was utilized in MS/MS fragmentation. The ions were discovered using a full-scan method with a mass range of 100 to 1500 *m/z* [67].

### 3.5. Volatile Compounds in Purslane

The volatile compounds in purslane extract were isolated and filtered using hydro-distillation. In total, 100 g of purslane whole plant powder were extracted with 800 mL of deionized water in a round bottom flask with a Clevenger-style setup under 70 °C for 24 h, the same method used as Politeo et al. [83]. The volatile compounds were homogenized with anhydrous MgSO_4_ to remove excess water. After settling, the magnesium salt was suspended [84].

In total, 10 µL of isolated volatile compounds (VOCs) were injected into an Aligant GC (Agilent Technologies, Santa Clara, CA, USA) equipped with a separation column (60 × 0.25 mm, 0.25 μm). The column was heated to 250 °C for 3 min to desorb the volatile compounds. Firstly, the temperature was adjusted at 40 °C for 3 min, then increased at a rate of 5 °C/min to 235 °C for 10 min. The mobile phase was helium gas with a 1.8 mL/min flow rate, and the detector was adjusted at 250 °C and 70 eV. The mass spectrum range was in the range of 40–450 mAU. The mass spectra of VOCs were identified compared to the NIST database, and the % area of the obtained peaks was calculated [85].

### 3.6. Purslane Extract Activity

#### 3.6.1. Scavenging Activity of PuE

The scavenging ability of purslane extract was determined according to Jia et al. [86]. In total, 50 µL of each PuE concentration (50, 150, 200, 250, 300, and 350 µg/mL) were loaded in the respective wells of the microtiter plate, and then 100 µL of ethanolic DPPH were added to each well; the microplate was kept in the dark for 30 min. The developed color was read at 515 nm using a microplate reader. The color change from purple to yellow correlated to the antioxidant activity of PuE against DPPH was calculated in Equation (2). The SC_50_ was calculated as the lowest concentration of PuE scavenged with 50% of DPPH free radicals.

(2) $$\%\;\mathrm{Scavenging}\;\mathrm{activity}=\frac{\mathrm{AC}-\mathrm{AS}}{\mathrm{AC}}\times 100,$$

where AC is control absorbance, and AS is sample absorbance.

#### 3.6.2. Cytotoxicity

Human breast (MCF-7), human colon (HCT-16), and human cervix (HeLa) cancer cell lines were obtained from the Biodiagnostic Labs (Dokki, Egypt). The in vitro antiproliferative activity of PuE was determined by measuring 3-(4,5-dimethylthiazol-2-yl)-2,5-diphenyltetrazolium bromide (MTT) dye absorbance in living cells compared to Doxorubicin (DOX) as a control. Briefly, cells were seeded in 96-well, flat-bottomed plates containing 100 μL OF a cell suspension with a known concentration per well and allowed to adhere at 37 °C in a humidified atmosphere containing 5% CO_2_. Usually, 50,000 cells were seeded per well. PuE (100 μL) were added to their respective wells at two concentrations (50 and 350 μg/mL) and Doxorubicin (DOX, 350 μg/mL). In total, 20 μL of the MTT solution (5 mg/mL; Sigma-Aldrich, St. Louis, MO, USA) were added at 24 h, 48 h, or 72 h for dyeing, and the cells were incubated for an additional 4 h at 37 °C. After incubation, the cell suspensions were centrifuged at 800 rpm for 10 min, and 100 μL of distilled water replaced the supernatants to solubilize the formazan crystals formed in viable cells. Absorbance at 570 nm was measured using a microplate ELISA reader. The results were expressed as a percentage of control proliferation (100%). The IC_50_ value was described as the concentration of PuE that inhibited the growth of cells by 50% [87].

The apoptosis-inducing potential of PuE was assessed by measuring the caspase-3 activity after treating the tested cancerous cells (2 × 10^6^ cells) with PuE or distilled water as a negative control at the proper doses and times. The cells’ lysates were collected by cold centrifugation at 300× *g* for 10 min, then incubated with caspase-3 substrate bisamide (Z-DEVD-R110) in a microplate reader for 45 min; the resultant fluorescence was observed, and the excitation and emission were measured at 485/530 nm [88].

#### 3.6.3. Antiviral Activity

The SARS-CoV2 Inhibitor Screening Assay kit (Adipogen, San Diego, CA, USA) was employed to block the interaction between Spike and angiotensin-converting enzyme-2 (ACE2). Spike receptor binding domain (RBD) (100 µL) was added into the plate wells; the plate was stored at 4 °C for 16 h. In total, 100 µL of PuE concentrations (50, 150, 200, 250, 300, and 350 µg/mL) were added to Spike wells and incubated (incubator IN55, Memmert, Germany) at 37 °C for 60 min in the presence of Inhibitor Mix Solution to assess the antiviral activity of PuE extract against SARS-CoV2. Human cells were used to generate ACE2 as a recombinant protein. After incubation, an HRP-labeled streptavidin was added to each well and incubated at room temperature for 60 min. The reaction was deactivated by adding tetramethyl benzidine (100 µL) for 5 min. The absorbance was measured using a microplate reader at 450 nm.

#### 3.6.4. Antimicrobial Activity

The antimicrobial activity of PuE concentrations (50, 150, 200, 250, 300, and 350 µg/mL) was performed against the tested pathogenic bacteria [(*Listera moncytogenesis* (LM), *Staphylococcus aureus* (SA), and *Escherichia coli* (EC), *Klebsiella pneumonia* (KP)], and fungi [*Candida albicans* (CA), and *Candida gelberta* (CG)]. These strains were selected based on the microbial count of spoiled yogurt samples. It was found during microbial examination with a light microscope and biochemical and morphological definitions that these isolates are the most isolates that cause yogurt spoilage. These isolates were confirmed by identification at the gene level through isolating DNA and using PCR to detect genes. The bacterial isolates were identified based on 16S rRNA and the fungal isolates on 18S rRNA gene sequence analysis. Genomic DNA was obtained by the hexadecyltrimethylammonium bromide (CTAB) technique, and the integrity and level of purified DNA were established by agarose gel electrophoresis. The DNA level was customized to 20 ng/µl for PCR amplification. The forward primer used with the isolates is (5 AGA GTT TGA TCC TGG CTC AG 3), and the reverse is (5 GGT TAC CTT GTT ACG ACT T 3). PCR products were isolated by electrophoresis on 1.5% agarose gels stained with ethidium bromide and documented in the Alphaimager TM1200 documentation and analysis system. The obtained polymorphic differences were analyzed via the program NTSYS-PC2 by assessing the distance of isolates by Jaccard’s Similarity Coefficient.

The tested strains were cultivated in a shaker incubator at 37 °C for 12 h for a concentration of (1 × 10^8^ CFU/mL). Muller Hinton broth (MHB) was used for bacteria, and Sabouraud dextrose broth for *Candida.* The analysis of antimicrobial activity was performed using the disc diffusion assay [89]. The bacteria and *Candida* inoculum (100 µL) were spread over Petri plates then paper discs (6 mm) previously saturated in 10 mL of PuE (50, 150, 200, 250, 300, and 350 µg/mL) were placed on the surface of the Muller Hinton agar (MHA) plates. The MHA plates were incubated at 37 °C for 24 h. A ruler was utilized to calculate the inhibition zones (mm). The positive controls, Levofloxacin and clotrimazole (Canesten) at 350 µg/mL, were used to compare the antimicrobial results against bacteria and fungi.

### 3.7. Experimental Safety Layout

#### 3.7.1. Ethical Statement

The animal study was reviewed and approved by the ZU-IACUC committee that was performed following the guidelines of the Egyptian Research Ethics Committee and the guidelines specified in the Guide for the Care and Use of Laboratory Animals (2023), ethical code number ZU-IACUC/2/F/398/2023. Written informed consent was obtained from the owners for the participation of their animals in this study.

Thirty Wister white rats (150–175 g) were acquired and randomly divided into five groups of six animals (Table 9).

The duration of the layout was four weeks. Rats were slain adequately at the end of the experiment, and liver and kidney specimens were obtained surgically. The retro-orbital vein was used to collect blood samples. The retro-orbital vein was used to gather blood samples to estimate the biochemical parameters of the blood.

#### 3.7.2. Estimation of Serum Biochemical Parameters

Blood samples were taken by retro-orbital vein using a 5 mL syringe after animals had fasted overnight and centrifuged at 900× *g* for 10 min to separate serum. The following serum biochemical parameters: total protein (TP), globulin (GL), albumin (ALB), total cholesterol (TC), low-density lipoprotein (LDL), high-density lipoprotein (HDL), triglyceride (TG), alanine aminotransferase (ALT), and aspartate aminotransferase (AST) were measured in serum using commercial biodiagnostic kits provided from Biodiagnostic Company (El-Tahrir St. Dokki, Giza, Egypt). These parameters were colorimetrically measured using a spectrophotometer (Shimadzu, Japan).

Schumann and Klauke [90] estimated the values of ALT, AST, total protein, and albumin. Beutler’s technique [91] was used to quantify serum-reduced glutathione. According to Beutler [91], Malondialdehyde (MDA) was computed as the reactive of thiobarbituric acid (TBA) as indicators of renal function, and urea and creatinine were assessed [92]. The total cholesterol, LDL, and HDL values were determined using an enzymatic colorimeter [93]. Total triglycerides were calculated using the method of Devi and Sharma [94].

#### 3.7.3. Protective Effect of Purslane Extract against Fe^2+^—Induced Brain Lipid Peroxidation

Fe^2+^-induced brain lipid peroxidation was performed by the method described by Oboh et al. [62]. A white Wister rat was anesthetized by diethyl ether and sacrificed; the cerebral tissue was rapidly separated, weighed, and kept in ice. The tissue was homogenized in cold saline solution (1% NaCl, *w/v*), and then the homogenate solution was centrifuged (SIGMA 3-30K, GmbH, Germany) at 7000 rpm for 10 min. The pellet was discarded, and the supernatant (S1) containing water, proteins, lipids (phospholipids, cholesterol, gangliosides, galactolipid), RNA, and DNA was collected for lipid peroxidation assay. In total, 50 µL of S1 fraction were mixed with a reaction mixture containing 15 µL of HCl buffer solution, different volumes of purslane extract (20, 40, or 50 µL), and 15 µL of freshly prepared FeSO_4_ solution, then completed to 150 µL by DW before incubation at 37 °C for 60 min. After adding 150 µL of SDS, the reaction color was developed. To the reaction mixture, 300 µL of acetic acid/HCl mixture and 300 µL of thiobarbituric acid solution were added and incubated at 100 °C for 60 min. The liberated reactive species of Thiobarbituric acid were measured at 532 nm using a microplate reader. The same procedure was used to prepare the control without extract. The percentage inhibition of Fe^2+^-induced lipid peroxidation was calculated using the following equation.

(3) $$\%\;\mathrm{inhibition}=\frac{\mathrm{AC}-\mathrm{AS}}{\mathrm{AC}}\times 100,$$

where AC is the control absorbance, and AS is the sample absorbance.

### 3.8. Preparation of Functional Yogurt Supplemented with Purslane Extract

Christian Hansen’s lab created the starting culture with equal weight (1 g) of each freeze-dried prophylactic, “*Lactobacillus acidophilus* (1.5 × 10^8^ CFU/g), *Bifidobacterium lactis* (1.0 × 10^7^ CFU/g)*,* and *Streptococcus thermophilus”* (1.5 × 10^7^ CFU/g). The final concentration of starter 1% was added to the milk to process the fermentation.

One liter of buffalo milk was provided from the local market with approximate composition: protein (3.9%), lactose (4.5%), ash (0.69%), total solids (13%), and solid not fats (8.5%). For pasteurization, the milk was heated to 85 °C for 45 min and then cooled to 42.5 °C to prepare the yogurt. The following components were added to the milk; the starting culture was (1%), 50 mL of PuE at three concentrations (50, 150, and 250 µg/mL), and sugar at 10% (*w/v*). The components were homogenized for 60 seconds using an electric stirrer (Braun MultiQuick9, Melsungen, Germany). The fermentation continued at 42.5 °C for 2 h until a hard curd formed [28] by measuring pH equal to 4.5, then the yogurt was chilled to 4 °C and stored. Table 1 shows the yogurt drink constituents. The purslane extract was rich in polysaccharides; therefore, we did not add sugar to purslane-enriched yogurt; it was added as a sweetener [68] (Supplementary Table S3).

### 3.9. Chemical Composition of Yogurt

The moisture, ash, crude protein, and fat levels of yogurt were determined by procedures 926.08, 942.05, 991.20, and 2000–18, respectively, according to AOAC [78]. The dietary fiber content was determined using the enzymatic approach developed by Moczkowska et al. [79]. The difference was measured by the carbohydrate content using Equation (1).

### 3.10. Fluctutaion of Purslane Extract—Yogyrt Properties during Four Weeks of Cold Storage

#### 3.10.1. Physicochemical Properties

##### pH Determination

Ten grams of yogurt samples were homogenized in 90 mL of water for 30 min using a magnetic stirrer and then filtrated. The pH of yogurt filtrate was measured using the pH meter (pH 211 HANNA, Cluj, Romania).

##### Titratable Acidity (TTA)

The TTA was determined using standard procedure 942.15 AOAC [78]. In brief, 10 g of yogurt samples were diluted with 90 mL of water, then titrated with NaOH (0.1 N) and phenolphthalein indicator. The TTA in yogurt was represented as % lactic acid.

##### Total Soluble Solids (TSS)

An ABBE refractometer (VEE GEE Model C10, Thermofisher Scientific, USA) measured the total soluble solids (TSS) by drops of yogurt drink placed on the refractometer glass front.

##### Malondialdehyde (MDA) Determination

Lipid peroxidation was evaluated at intervals of 0, 7, 14, 21, and 30 days of storage at 4 °C of experimental yogurts prepared with PuE (50, 150, and 250 µg/mL) by measuring MDA according to the method of Guemidi et al. [73]. Ten grams of yogurt were put in glass tubes containing ice to prevent oxidation, and then 100 µL of ascorbic acid and 20 mL of a TCA (5%, *w*/*v*) were added. The mixture was homogenized and then centrifuged (SIGMA 3-30K, GmbH, Germany) at 10,000 rpm for 10 min; then, the supernatant was collected, and 2 mL from the supernatant was added to 2 mL of TBA (20 mmol/L) in tubes of 15 mL, which were then heated for 15 min in a 100 °C water bath and cooled to room temperature. A spectrophotometer (JANWAY 7205, UK) measured the absorbance at 532 nm against the control of 2 mL TBA and 2 mL of TCA. Absorbance was applied using the following equation:

(4) $$\mathrm{mg}\frac{\mathrm{MDA}}{\mathrm{kg}}=\left(A532\times \mathrm{VTCA}\times \mathrm{Vf}\right)\times \left(\frac{0.76}{1.56}\right)\times m,$$

where A532 is the absorbance at 532 nm; VTCA, 20 mL of TCA; Vf, supernatant volume; m, sample weight; 0.76/1.56 MDA/TBA molecular extinction coefficient.

##### Fat Content

The Gerber method identified the fat level. In total, 10 mL of yogurt filtrate were added into the butyrometer, then 10 mL of sulphuric acid and 1 mL of amyl alcohol were added; the butyrometer was closed and shacked until no white particles were seen, then put in a water bath for 5 min. The Gerber tubes were centrifuged at 1200 rpm for 4 min and then placed in the water bath. The fat percentage was calculated by reading the bottom of the fat column to the lower border of the meniscus on the scale.

The fat content should range between 0.37 and 4% while retaining the protein’s impacts on texture, stability, and perceived viscosity.

##### Syneresis

Syneresis was determined by whey separation. Ten grams of yogurt samples were centrifuged at 10,000 rpm for 5 min, the supernatant was collected, and syneresis was calculated from the equation [74].

(5) $$\mathrm{Synersis}=\frac{m\;\mathrm{supernatent}}{m\;\mathrm{yogurt}}\times 100,$$

##### Viscosity

Ten grams of yogurt were evaluated using a Rheometer (ARES-G2, TA instruments, New Castle, DE, USA) at 4 °C. The rheometer was coupled with a spindle at 4–100 rpm and 40–50% torque. The yogurt’s viscosity was measured in centipoise units (cP) [95].

##### Water Holding Capacity (WHC)

Ten grams of yogurt samples were homogenized in 90 mL of water, stirred for one hour, and then centrifuged at 3000 rpm for 20 min. The supernatant was discarded, and the residue was weighed, then dried at 120 °C for one hour and reweighed. WHC was calculated as mL of retained water/g of the sample [96].

##### Texture Profile Analysis

An extruder (Stable Micro Systems, Godalming, UK) equipped with a 35 mm disc was used to analyze the textural characteristics of yogurt samples. The velocity was 1 mm/s, the target distance was 30 mm, and the trigger weight was 10 g. The hardness, adhesiveness, and consistency values were assessed for all samples at 0, 7, 14, 21, and 30 days, following the method of Nguyen et al. [97].

#### 3.10.2. Determination of Antioxidant Activity, Phenolic Content in Yogurt Samples

The antioxidant content in yogurt samples was determined as mentioned in Section 3.4.1 and Section 3.6.1.

#### 3.10.3. Sensorial Traits and Color Measurements

Ten trained panelists, six men and four women aged between 40 and 55, evaluated the sensory qualities of yogurt samples (flavor, color, texture, shape, and all-over acceptability) using nine hedonic scales where “nine” was liked extremely and “one” was disliked extremely. Yogurt samples (50 g) were presented in cups coded with three digits; each participant was given water to remove the effects of each tested sample, which was then evaluated accordingly [28].

The color parameters and the color change ΔE of yogurt samples were examined with a Hunter spectrophotometer (Color Flex EZ’s, Reston, VA, USA) and calculated following the method of Namir et al. [98].

(6) $$\Delta E=\sqrt{\Delta L^{2}}+\Delta a^{2}+\Delta b^{2},$$

where L is lightness (100) to darkness (0); (a) redness (+) to greenness (−); b yellowness (+) to blueness (−).

#### 3.10.4. Total Viable and Lactic Acid Bacteria Count

Ten grams of yogurt samples (control and supplemented) were homogenized in 90 mL of peptone buffer water to create yogurt suspension. Then, 10^−1^–10^−8^ decimal dilutions were prepared. The dilutions were added to specific media plates [99]. Lactic acid bacteria (LAB) were counted on the MRS medium after three days of incubation at 37 °C [28]. The total counts (CFU/g) of microbial results were converted to logarithms.

### 3.11. Statistical Analysis

The triplicate findings were analyzed using ANOVA to investigate the variability between the data means at the confidence level of 95%. The post hoc test, LSD, was used to compare the data average to elucidate the significant differences at a 5% probability level. SPSS 23.0 software (IBM, Armonk, New York, NY, USA) was used for the statistical analysis.

## 4. Conclusions

Purslane whole plant extract is rich in polyphenols, flavonoids, and volatile compounds; therefore, it exhibits high antimicrobial activity against pathogenic bacteria and *Candida*. Furthermore, purslane extracts demonstrate excellent scavenging, cytotoxicity, and antiviral properties; therefore, supplementing yogurt with purslane extract at three concentrations (50, 150, and 250 µg/g) significantly enhances antioxidant capacity and storage stability. The PuE-yoghurt supplemented with purslane extract at 150 µg/g recorded high scores of color, texture, and sensory properties. This study suggested that fortifying yogurt with PuE of 150 µg/g improved natural functional foods’ scavenging activity and stability. *P. oleracea* might be utilized in functional foodstuffs because it contains bioactive compounds and is grown inexpensively worldwide. Thus, food manufacturers might utilize it for functional food manufacturing.

## Figures and Tables

**Table 1:** Proximate analysis and phytochemicals content of whole purslane.

Parameters	Concentration (%)
Moisture	10.9 ± 0.2
Total ash	2.45 ± 0.5
Crude fiber	21.63 ± 0.8
Crude protein	18.2 ± 0.1
Crude fat	5.2 ± 0.4
Carbohydrates	44.3 ± 0.6
Minerals	Concentration (ppm)
Mg	695 ± 0.9 ^b^
Ca	671 ± 1.1 ^b,c^
P	450 ± 0.8 ^c^
K	5033 ± 2.1 ^a^
Fe	20 ± 0.5 ^d^
Phytochemicals	Concentration (mg/mL)
Phenolic compounds	250 ± 1.2
Flavonoids	56 ± 0.9

*n* = 3, The statistics are displayed as the mean ± standard deviation. Different lowercase letters (a–d) in the same column indicate significant differences between mineral content using the LSD test at *p* < 0.05.

**Table 2:** High-resolution profile of phenolic compounds in *P. oleracea* extract detected by LC/MS.

RT	[M-H]^−1^ Ions at m/z	% Base Peak (MS^2^)	Identified Compounds	Contents (mg/100 g)
Phenolic acids				
3.25	311	151	protocatechuic acid glu	8.55 ± 0.1
5.00	472	132, 151, 312	protocatechuic acid caffeoyl-glu	11.36 ± 0.3
9.00	135	115	p-hydroxybenzoic acid	12.89 ± 0.9
10.44	351	190	chlorogenic acid	16.56 ± 0.5
10.55	339	133, 177	caffeic acid glu	30.69 ± 0.8
11.30	322	165	coumaric acid glu	18.21 ± 0.2
14.18	381	222	sinapic acid glu	10.35 ± 0.3
16.30	350	190	neochlorogenic acid	21.64 ± 0.4
18.55	335	165	p-coumaroylquinic acid	14.33 ± 0.6
20.22	195	165	syringic acid	12.69 ± 0.1
*p*-value				0.025
Flavonoids				
13.51	302	195	gallo-catechin	25.63 ± 0.2
22.79	287	243	catechin	29.65 ± 0.6
28.52	440	303	quercetin rham	15.23 ± 0.1
28.62	444	286	kaempferol glu	16.54 ± 0.2
31.22	459	282	kaempferol glca	15.33 ± 0.9
31.78	415	266	naringenin rham	10.68 ± 0.3
*p*-value				0.029
Tannins				
3.71	329	122, 168	galloyl glucose	11.2 ± 0.2
7.50	338	133, 177	caffeoyl glucose	9.6 ± 0.6
13.88	351	192	feruloyl glucose	3.5 ± 0.5
15.79	428	312	coumaric galloyl-malate	3.1 ± 0.3
16.01	423	311	benzyl-o-galloyl glucose	8.9 ± 0.9
*p*-value				0.031
Phenolic derivatives				
23.95	395	195	syringic acid derivatives	5.6 ± 0.0
24.25	445	265	apigenin derivatives	10.7 ± 0.2
*p*-value				0.019
Organic acids				
1.36	190	110	quinic acid	510 ± 1.1
1.51	132	–	malic acid	550 ± 2.3
2.62	190	111	citric acid	590 ± 0.9
*p*-value				0.05

Retention time (RT, min), glucoside (Glu), rhamnoside (Rha), glucuronide (GlcA), *n* = 3, values are presented mean ± SD.

**Table 3:** GC-MS profile of volatile compounds (% dry matter DM) detected in *P. oleracea* extract by.

VOCs	LRI	% DM
Oxygenated compounds		
hexanal	810	0.43
heptanal	903	0.56
benzaldehyde	960	1.35
nonanal	1100	1.2
(*E*)-2-octenal	1060	1.33
safranal	1205	1.22
cuminal	1240	2.2
(*E*,*Z*)-3,5-octadien-2-one	1070	0.65
camphor	1150	1.02
(*E*)-2-octen-1-ol	1066	1.12
Nitrogen compounds		
2,3-dimethyl pyrazine	915	0.5
2,3,5-trimethyl pyrazine	917	0.5
Terpenoids		
β-cyclocitral	1224	2.25
limonene	1032	30.12
linalool	1101	2.15
menthone	1155	1.1
menthol	1175	1.2
carvone	1244	41.63
β-caryophyllene	1420	2.1
(*E*)-β-ionone	1487	0.65
Hydrocarbons		
2-pentyl furan	995	1.3
2,6-dimethylcyclohexanol	1110	5.2
Oxygenated compounds		11.8%
Nitrogen compounds		1%
Terpenoids		81.2%
Hydrocarbons		6.5%

Liner retention index (LRI), volatile compounds (VOCs); *n* = 3.

**Table 4:** Inhibition zones diameters of *P. oleracea* extract with different concentrations against pathogenic bacteria and *Candida* compared to a bacterial and fungal antibiotic after day incubation at 37 °C.

Microorganisms	Purslane Extract Concentration (µg/mL)/Inhibition Zones (cm)						Levofloxacin	MIC
Bacteria	50	150	200	250	300	350	350 µg/mL	
LM	1.2 ± 0.0 ^c^	1.4 ± 0.2 ^d^	1.7 ± 0.2 ^d^	2.1 ± 0.2 ^d^	2.7 ± 0.3 ^c^	3.9 ± 0.2 ^b^	3.7 ± 0.2 ^b^	30 ^c^
SA	1.6 ± 0.1 ^a^	2.0 ± 0.6 ^a^	2.4 ± 0.1 ^a^	2.9 ± 0.2 ^a^	3.2 ± 0.1 ^a^	4.2 ± 0.0 ^a^	3.9 ± 0.1 ^a^	20 ^e^
EC	1.1 ± 0.2 ^c,d^	1.3 ± 0.1 ^d,e^	1.6 ± 0.3 ^d,e^	1.9 ± 0.3 ^e^	2.4 ± 0.2 ^d^	3.0 ± 0.1 ^d^	2.9 ± 0.4 ^d^	35 ^b^
KP	1.0 ± 0.3 ^c^	1.2 ± 0.6 ^e^	1.5 ± 0.1 ^e^	1.8 ± 0.2 ^e^	2.3 ± 0.6 ^d^	2.8 ± 0.2 ^e^	2.7 ± 0.7 ^e^	40 ^a^
Fungi	50	150	200	250	300	350	Canesten	MIC
CG	1.2 ± 0.1 ^c^	1.6 ± 0.3 ^c^	2.0 ± 0.5 ^c^	2.3 ± 0.4 ^c^	2.6 ± 0.1 ^cd^	3.1 ± 0.2 ^d^	2.9 ± 0.2 ^d^	35 ^b^
CA	1.4 ± 0.2 ^b^	1.8 ± 0.5 ^b^	2.3 ± 0.6 ^b^	2.5 ± 0.8 ^b^	3.0 ± 0.0 ^b^	3.3 ± 0.3 ^c^	3.1 ± 0.1 ^c^	25 ^d^

*Listera moncytogenesis* (LM), *Staphylococcus aureus* (SA), *Escherichia coli* (EC), *Klebsiella pneumonia* (KP), *Candida albicans* (CA), and *Candida gelberta* (CG). Data are presented as the mean of triplicates ± SD. Lowercase letters (a–e) in the same column indicate a significant difference between the effect of *P. oleracea* extract on tested bacteria and fungi using the LSD test at *p* < 0.05.

**Table 5:** The effect of *P. oleracea* extract (PuE) in Wister rat diet on the liver, kidney parameters, and lipid profile in Wister rats compared to BHA.

Blood Parameters	Control	BHA	PuE (µg/g)		
Liver Parameters		250 µg/g	50	150	250
ALT	43.8 ± 0.2 ^c^	88.2 ± 0.2 ^a^	53.6 ± 0.2 ^b^	50.6 ± 0.1 ^b^	52 ± 0.2 ^b^
AST	35.6 ± 0.6 ^c^	81.6 ± 0.1 ^a^	45.1 ± 0.3 ^b^	40.2 ± 0.6 ^b,c^	43 ± 0.3 ^b,c^
MDA	46.36 ± 0.9 ^c^	67.9 ± 0.3 ^a^	51.52 ± 0.1 ^b^	50.51 ± 0.1 ^b^	50.9 ± 0.1 ^b^
GSH	57.5 ± 0.1 ^a^	40.2 ± 0.4 ^c^	51.2 ± 0.4 ^b^	56.3 ± 0.2 ^a^	53 ± 0.6 ^a^
TP	6.14 ± 0.7 ^a^	5.61 ± 0.9 ^b^	5.98 ± 0.7 ^b^	6.22 ± 0.8 ^a^	6.02 ± 0.6 ^a,b^
Kidney parameters					
Urea	16.6 ± 0.2 ^c^	27 ± 0.3 ^a^	19.2 ± 0.1 ^b^	17.4 ± 0.2 ^c^	18.1 ± 0.2 ^b,c^
Creatinine	0.39 ± 0.001 ^d^	0.82 ± 0.04 ^a^	0.55 ± 0.02 ^b^	0.46 ± 0.01 ^c^	0.51 ± 0.1 ^b,c^
Lipid profile					
LDL	16.7 ± 0.3 ^c^	85.4 ± 0.5 ^a^	24.9 ± 0.3 ^b^	23.5 ± 0.1 ^b^	24.0 ± 0.3 ^b,c^
HDL	36.2 ± 0.4 ^a,b^	25.4 ± 0.2 ^c^	37.3 ± 0.4 ^a^	34.2 ± 0.5 ^b^	37.9 ± 0.5 ^a^
TG	77.5 ± 0.9 ^c^	119.2 ± 0.9 ^a^	84 ± 0.1 ^b^	79.6 ± 0.6 ^c^	81.3 ± 0.6 ^b,c^
TC	67.4 ± 0.1 ^c^	135.2 ± 0.8 ^a^	77.1 ± 0.9 ^b^	72.6 ± 0.9 ^c^	75.4 ± 0.7 ^b,c^

PC rats fed BHA supplemented diet (positive control); PuE-rats received a diet supplemented with PuE (50, 150, and 250 µg/g). The findings are presented as mean ± standard deviation; lowercase letters (a–d) in each column indicate significant differences between PuE concentration and BHA in rat diet using the LSD at *p* < 0.05.

**Table 6:** Physicochemical properties of yogurt supplemented with *P. oleracea* extract (50, 150, and 250 µg/g) during the experimental period of 30 days (mean ± SD).

Yogurt		Period (Days)	pH	Acidity (mg/10 mL)	Fat (%)	TSS (%)	Syneresis (%)	Viscosity (cP)	WHC (mL/g)
PuE concentration (µg/g)	Control (0)	0	4.52 ± 0.0 ^a,b^	80.15 ± 0.2 ^f^	1.33 ± 0.1 ^f^	11.99 ± 0.3 ^g^	9.2 ± 0.2 ^d,e^	154.3 ± 0.6 ^i^	7.2 ± 0.1 ^d^
		7	4.42 ± 0.2 ^c^	82.66 ± 0.1 ^e^	1.45 ± 0.1 ^e,f^	12.11 ± 0.1 ^f^	9.4 ± 0.1 ^d^	150.2 ± 0.8 ^i^	7.0 ± 0.5 ^d,e^
		14	4.35 ± 0.3 ^d^	86.25 ± 0.9 ^c^	1.62 ± 0.0 ^d^	12.46 ± 0.4 ^e^	9.9 ± 0.6 ^c^	145.7 ± 0.7 ^j^	6.4 ± 0.3 ^e^
		21	4.22 ± 0.1 ^f^	88.47 ± 0.4 ^b^	1.77 ± 0.2 ^c^	12.69 ± 0.6 ^d^	10.4 ± 0.8 ^b^	142.6 ± 0.5 ^j^	6.2 ± 0.1 ^e,f^
		30	4.11 ± 0.2 ^g^	90.25 ± 0.8 ^a^	1.89 ± 0.1 ^c^	12.78 ± 0.1 ^c^	11.0 ± 0.4 ^a^	139.4 ± 0.8 ^k^	5.9 ± 0.8 ^f^
	50	0	4.50 ± 0.1 ^b^	81.33 ± 0.7 ^e^	1.45 ± 0.5 ^e,f^	12.00 ± 0.2 ^f,g^	8.9 ± 0.7 ^e^	198.2 ± 0.1 ^g^	7.7 ± 0.6 ^d^
		7	4.45 ± 0.0 ^b,c^	82.25 ± 0.3 ^e^	1.67 ± 0.4 ^d^	12.35 ± 0.1 ^f^	9.2 ± 0.5 d^e^	189.1 ± 0.8 ^g^	7.5 ± 0.4 ^d^
		14	4.36 ± 0.2 ^d^	84.99 ± 0.9 ^c,d^	1.88 ± 0.3 ^c^	12.56 ± 0.4 ^e^	9.4 ± 0.6 ^d^	180.3 ± 0.9 ^h^	7.3 ± 0.2 ^d^
		21	4.29 ± 0.1 ^e^	86.78 ± 0.4 ^c^	1.92 ± 0.9 ^b^	12.75 ± 0.9 ^c^	9.7 ± 0.5 ^c^	177.6 ± 08 ^h^	7.0 ± 0.3 ^d,e^
		30	4.21 ± 0.1 ^f^	89.14 ± 0.5 ^a,b^	1.99 ± 0.1 ^b^	12.89 ± 0.4 ^b,c^	10.2 ± 0.3 ^b^	165.5 ± 0.3 ^i^	6.8 ± 0.0 ^e^
	150	0	4.55 ± 0.0 ^a^	80.92 ± 0.2 ^f^	1.51 ± 0.0 ^e^	12.22 ± 0.2 ^f^	8.1 ± 0.1 ^f^	275.3 ± 1.2 ^d^	8.4 ± 0.1 ^b,c^
		7	4.50 ± 0.2 ^b^	81.05 ± 0.3 ^e^	1.79 ± 0.3 ^c^	12.51 ± 0.3 ^e^	8.3 ± 0.8 ^f^	262.1 ± 1.1 ^d,e^	8.2 ± 0.2 ^c^
		14	4.41 ± 0.3 ^c^	83.98 ± 0.1 ^d^	1.96 ± 0.2 ^b^	12.79 ± 0.1 ^c^	8.6 ± 0.3 ^e^	259.3 ± 1.1 ^e^	8.0 ± 0.3 ^c^
		21	4.36 ± 0.0 ^d^	85.24 ± 0.2 ^c,d^	2.07 ± 0.1 ^a,b^	12.98 ± 0.2 ^b^	9.1 ± 0.3 ^d,e^	241.6 ± 0.9 ^e,f^	7.5 ± 0.1 ^d^
		30	4.30 ± 0.2 ^e^	88.21 ± 0.1 ^b^	2.11 ± 0.2 ^a^	13.25 ± 0.3 ^a^	9.4 ± 0.6 ^d^	239.5 ± 0.5 ^f^	7.2 ± 0.7 ^d^
	250	0	4.53 ± 0.1 ^a,b^	81.21 ± 0.7 ^e,f^	1.49 ± 0.5 ^e^	12.11 ± 0.5 ^f^	7.5 ± 0.7 ^g^	345.2 ± 0.9 ^a^	9.3 ± 0.6 ^a^
		7	4.49 ± 0.3 ^b^	82.00 ± 0.8 ^e^	1.78 ± 0.6 ^c^	12.46 ± 0.2 ^e^	7.8 ± 0.1 ^g^	331.4 ± 0.8 ^a,b^	9.1 ± 0.1 ^a,b^
		14	4.40 ± 0.0 ^c^	84.51 ± 0.7 ^d^	1.92 ± 0.4 ^b^	12.62 ± 0.1 ^d^	8.1 ± 0.2 ^f^	320.7 ± 0.4 ^b^	8.8 ± 0.6 ^b^
		21	4.32 ± 0.2 ^e^	86.00 ± 0.9 ^c^	1.99 ± 0.5 ^b^	12.87 ± 0.3 ^b,c^	8.5 ± 0.3 ^e,f^	310.9 ± 0.6 ^c^	8.6 ± 0.3 ^b^
		30	4.23 ± 0.1 ^f^	88.95 ± 0.0 ^b^	2.05 ± 0.4 ^a,b^	13.00 ± 0.1 ^a,b^	8.8 ± 0.4 ^e^	305.3 ± 0.5 ^c^	8.5 ± 0.4 ^b,c^

Lowercase letters (a–k) in each column indicate significant differences in the physicochemical properties of yogurt affected by *P. oleracea* extract concentrations at *p* ≤ 0.05 using the LSD test.

**Table 7:** MDA content (mg/kg) in yogurt samples indicates lipid oxidation during yogurt preservation for 30 days.

Yogurt Samples	Storage Period				
	0 d	7 d	14 d	21 d	30 d
Control	0.92 ± 0.01 ^a^	1.01 ± 0.03 ^a^	1.23 ± 0.02 ^a^	1.31 ± 0.05 ^a^	1.77 ± 0.01 ^a^
PuE 50	0.88 ± 0.00 ^b^	0.93 ± 0.06 ^b^	1.05 ± 0.04 ^b^	1.21 ± 0.01 ^b^	1.35 ± 0.06 ^b^
PuE 150	0.81 ± 0.01 ^c^	0.89 ± 0.03 ^c^	0.93 ± 0.08 ^c^	0.97 ± 0.02 ^c^	1.05 ± 0.04 ^c^
PuE 250	0.83 ± 0.02 ^c^	0.91 ± 0.03 ^c^	1.02 ± 0.01 ^d^	1.19 ± 0.09 ^d^	1.26 ± 0.01 ^d^

Lowercase letters (a–d) in each column indicate significant differences in MDA content in yogurt affected by *P. oleracea* extract concentrations during cold storage of 30 d at *p* ≤ 0.05 using the LSD test.

**Table 8:** Sensory properties of *P. oleracea* extract-yogurt (50, 150, and 250 µg/g) during cold preservation (mean ± SD).

Yogurt Samples		Storage (Days)	Color	Flavor	Texture	Taste	Overall Acceptability
PuE concentration (µg/g)	Control (0)	0	9.0 ± 0.0 ^a^	9.0 ± 0.0 ^a^	8.2 ± 0.0 ^c,d^	8.4 ± 0.2 ^a^	8.7 ± 0.0 ^b,c^
		7	8.7 ± 0.2 ^b,c^	8.4 ± 0.1 ^c^	8.2 ± 0.1 ^c,d^	8.1 ± 0.1 ^b,c^	8.4 ± 0.1 ^c,d^
		14	8.5 ± 0.3 ^cd^	8.1 ± 0.0 ^d^	8.0 ± 0.2 ^d^	7.9 ± 0.0 ^c^	8.2 ± 0.2 ^d^
		21	8.0 ± 0.2 ^e^	7.6 ± 0.2 ^e^	7.5 ± 0.1 ^e^	7.4 ± 0.1 ^e^	7.7 ± 0.3 ^e^
		30	7.5 ± 0.7 ^f^	7.5 ± 0.3 ^e^	7.0 ± 0.0 ^f^	6.9 ± 0.2 ^f^	7.3 ± 0.4 ^f^
	50	0	9.0 ± 0.0 ^a^	9.0 ± 0.0 ^a^	8.6 ± 0.0 ^b,c^	8.4 ± 0.1 ^a^	8.9 ± 0.0 ^a,b^
		7	8.8 ± 0.1 ^b^	8.7 ± 0.2 ^b,c^	8.5 ± 0.1 ^c^	8.2 ± 0.2 ^b^	8.7 ± 0.1 ^b,c^
		14	8.7 ± 0.2 ^b,c^	8.5 ± 0.1 ^c^	8.3 ± 0.2 ^c,d^	8.1 ± 0.1 ^b,c^	8.5 ± 0.0 ^c,d^
		21	8.5 ± 0.1 ^c,d^	8.4 ± 0.2 ^c^	8.0 ± 0.2 ^d^	7.9 ± 0.2 ^c^	8.3 ± 0.2 ^d^
		30	8.3 ± 0.1 ^d^	8.2 ± 0.1 ^c,d^	7.7 ± 0.1 ^f^	7.7 ± 0.1 ^d^	8.1 ± 0.1 ^d,e^
	150	0	9.0 ± 0.0 ^a^	9.0 ± 0.0 ^a^	8.9 ± 0.0 ^a^	8.4 ± 0.0 ^a^	9.0 ± 0.0 ^a^
		7	8.8 ± 0.0 ^b^	8.8 ± 0.1 ^b^	8.7 ± 0.1 ^b^	8.2 ± 0.2 ^b^	8.8 ± 0.1 ^b^
		14	8.6 ± 0.1 ^c^	8.7 ± 0.2 ^b,c^	8.5 ± 0.2 ^c^	8.1 ± 0.1 ^b,c^	8.6 ± 0.2 ^c^
		21	8.6 ± 0.1 ^c^	8.5 ± 0.1 ^c^	8.3 ± 0.1 ^c,d^	8.0 ± 0.1 ^c^	8.5 ± 0.1 ^c,d^
		30	8.5 ± 0.2 ^c,d^	8.3 ± 0.2 ^c,d^	8.0 ± 0.0 ^d^	7.9 ± 0.1 ^c,d^	8.3 ± 0.2 ^d^
	250	0	9.0 ± 0.0 ^a^	9.0 ± 0.0 ^a^	8.5 ± 0.2 ^c^	8.4 ± 0.4 ^a^	8.8 ± 0.1 ^b^
		7	8.7 ± 0.1 ^b,c^	8.5 ± 0.2 ^c^	8.4 ± 0.1 ^c^	8.1 ± 0.2 ^b,c^	8.5 ± 0.2 ^c,d^
		14	8.6 ± 0.2 ^c^	8.3 ± 0.3 ^c,d^	8.2 ± 0.3 ^c,d^	8.0 ± 0.1 ^c^	8.4 ± 0.1 ^d^
		21	8.2 ± 0.3 ^d^	8.1 ± 0.2 ^d^	8.0 ± 0.2 ^d^	7.6 ± 0.1 ^d^	8.1 ± 0.0 ^d,e^
		30	8.1 ± 0.2 ^d^	8.0 ± 0.1 ^d^	7.6 ± 0.1 ^f^	7.5 ± 0.2 ^d,e^	7.9 ± 0.2 ^e^

Lowercase letters (a–f) in each column indicate significant differences in sensory parameters in yogurt affected by *P. oleracea* extract concentrations during cold storage of 30 d at *p* ≤ 0.05 using the LSD test.

**Table 9:** Experimental design of purslane extracts safety and beneficial properties in Wister rats.

Treatments	Basel Diet (kg)	PuE (mg/kg)	BHA (mg/kg)
1	1	–	–
2	1	–	350
3	1	100	–
4	1	200	–
5	1	350	–

## Data Availability

Not applicable.

## Supplementary Materials

The following supporting information can be downloaded at: https://www.mdpi.com/article/10.3390/molecules28155859/s1, Figure S1: The impact of purslane extract at three concentrations (50, 150, and 250 µg/g) on yogurt texture properties (A, Firminess, B, consistency, C, adhesiveness) during cold storage.; Table S1: Growth parameters of Wister rats fed a diet supplemented with *P. oleracea* extract at three concentrations (50, 150, and 250) µg/g compared to BHA. Table S2: color parameters and color change during the storage of yogurt samples for 30 days. Table S3: The constituents of purslane extract-supplemented yogurt.

## Sample Availability

Samples of the purslane extract are available from the authors.

## Abbreviations

The purslane extract (PuE), breast cancer (MCF-7), colon cancer (HCT), and cervical cancer (HeLa) cell lines, butylated hydroxyanisole (BHA), high-density lipoprotein (HDL), low-density lipoprotein (LDL), aspartate aminotransferase (AST), alanine aminotransferase (ALT), malondialdehyde (MDA), total protein (TP), albumin (TA), and glutathione (GSH), total soluble solids (TSS), lactic acid bacteria (LAB), water-holding capacity (WHC), *Listera moncytogenesis* (LM), *Staphylococcus aureus* (SA), *Escherichia coli* (EC), *Klebsiella pneumonia* (KP), *Candida albicans* (CA), *Candida gelberta* (CG), trichloro acetic acid (TCA), tri barbituric acid (TBA), angiotensin-converting enzyme-2 (ACE2), 3-(4,5-dimethyl-thiazol-2-yl)-2,5-diphenyltetrazolium bromide (MTT), Doxorubicin (DOX), receptor binding domain (RBD), and inhibition zone diameters (IZDs).

## References

[1] Adinepour F., Pouramin S., Rashidinejad A., Jafari S.M. (2022). Fortification/enrichment of milk and dairy products by encapsulated bioactive ingredients. Food Res. Int..

[2] Minj J., Dogra S., Minj J., Sudhakaran V.A., Kumari A. (2020). Significance of Fortification of Beneficial Natural Ingredients in Milk and Milk Products. Dairy Processing: Advanced Research to Applications.

[3] Paswan V.K., Rose H., Singh C.S., Yamini S., Rathaur A. (2021). Herbs and spices fortified functional dairy products. Herbs and Spices—New Processing Technologies.

[4] Anbudhasan P., Surendraraj A., Karkuzhali S., Sathishkumaran S. (2014). Natural antioxidants and its benefits. Int. J. Food Nutr. Sci..

[5] Atta E.M., Mohamed N.H., Silaev A.A.A. (2017). Antioxidants: An overview on the natural and synthetic types. Eur. Chem. Bull..

[6] Venkatesh R., Sood D. (2011). A Review of the Physiological Implications of Antioxidants in Food.

[7] Kandylis P. (2022). Phytochemicals and antioxidant properties of edible flowers. Appl. Sci..

[8] de Carvalho A.P.A., Conte-Junior C.A. (2021). Health benefits of phytochemicals from Brazilian native foods and plants: Antioxidant, antimicrobial, anti-cancer, and risk factors of metabolic/endocrine disorders control. Trends Food Sci. Technol..

[9] Adebooye O.C., Vijayalakshmi R., Singh V. (2008). Peroxidase activity, chlorophylls and antioxidant profile of two leaf vegetables (*Solanum nigrum* L. and *Amaranthus cruentus* L.) under six pretreatment methods before cooking. Int. J. Food Sci. Technol..

[10] Saad A.M., Mohamed A.S., Ramadan M.F. (2021). Storage and heat processing affect flavors of cucumber juice enriched with plant extracts. Int. J. Veg. Sci..

[11] Lee Y.H., Choo C., Watawana M.I., Jayawardena N., Waisundara V.Y. (2015). An appraisal of eighteen commonly consumed edible plants as functional food based on their antioxidant and starch hydrolase inhibitory activities. J. Sci. Food Agric..

[12] Abd El-Hack M.E., El-Saadony M.T., Elbestawy A.R., Gado A.R., Nader M.M., Saad A.M., El-Tahan A.M., Taha A.E., Salem H.M., El-Tarabily K.A. (2022). Hot red pepper powder as a safe alternative to antibiotics in organic poultry feed: An updated overview. Poult. Sci..

[13] Côté J., Caillet S., Doyon G., Sylvain J.-F., Lacroix M. (2010). Bioactive compounds in cranberries and their biological properties. Crit. Rev. Food Sci. Nutr..

[14] El-Saadony M.T., Abuljadayel D.A., Shafi M.E., Albaqami N.M., Desoky E.-S.M., El-Tahan A.M., Mesiha P.K., Elnahal A.S., Almakas A., Taha A.E. (2021). Control of foliar phytoparasitic nematodes through sustainable natural materials: Current progress and challenges. Saudi J. Biol. Sci..

[15] Cosme P., Rodríguez A.B., Espino J., Garrido M. (2020). Plant phenolics: Bioavailability as a key determinant of their potential health-promoting applications. Antioxidants.

[16] Abd Elkader A.M., Labib S., Taha T.F., Althobaiti F., Aldhahrani A., Salem H.M., Saad A., Ibrahim F.M. (2022). Phytogenic compounds from avocado (*Persea americana* L.) extracts; antioxidant activity, amylase inhibitory activity, therapeutic potential of type 2 diabetes. Saudi J. Biol. Sci..

[17] Saad A.M., Salem H.M., El-Tahan A.M., El-Saadony M.T., Alotaibi S.S., El-Shehawi A.M., Abd El-Mageed T.A., Taha A.E., Alkahtani M.A., Ahmed A.E. (2022). Biological control: An effective approach against nematodes using black pepper plants (*Piper nigrum* L.). Saudi J. Biol. Sci..

[18] Salman K., Mahmoud E.A., Abd-Alla A. (2020). Preparing untraditional kishk formula with purslane as natural source of bioactive compounds. J. Food Dairy Sci..

[19] Salehi M., Sadeghi Mahoonak A., Khomeiri M. (2021). Fortification of yogurt with common purslane (*Portulaca oleracea*): Evalution of its fatty acid profile and antioxidant properties. J. Food Process. Preserv..

[20] Kumar A., Sreedharan S., Kashyap A.K., Singh P., Ramchiary N. (2021). A review on bioactive phytochemicals and ethnopharmacological potential of purslane (*Portulaca oleracea* L.). Heliyon.

[21] Silva R., Carvalho I.S. (2014). In vitro antioxidant activity, phenolic compounds and protective effect against DNA damage provided by leaves, stems and flowers of *Portulaca oleracea* (Purslane). Nat. Prod. Commun..

[22] Srivastava R., Srivastava V., Singh A. (2023). Multipurpose benefits of an underexplored species purslane (*Portulaca oleracea* L.): A critical review. Environ. Manag..

[23] Hussien H.A., Salem E.M. (2016). Development of gluten free snacks fortified with purslane (*Portulaca oleracea*) powder. Int. J. Food Sci. Nutr..

[24] Melilli M.G., Pagliaro A., Scandurra S., Gentile C., Di Stefano V. (2020). Omega-3 rich foods: Durum wheat spaghetti fortified with *Portulaca oleracea*. Food Biosci..

[25] Abd El-Azime A.S., Hussein E.M., Ashry O.M. (2014). Synergestic effect of aqueous purslane (*Portulaca oleracea* L.) extract and fish oil on radiation-induced damage in rats. Int. J. Radiat. Biol..

[26] Fontana L., Bermudez-Brito M., Plaza-Diaz J., Munoz-Quezada S., Gil A. (2013). Sources, isolation, characterisation and evaluation of probiotics. Br. J. Nutr..

[27] Ashaolu T.J. (2020). Immune boosting functional foods and their mechanisms: A critical evaluation of probiotics and prebiotics. Biomed. Pharmacother..

[28] El-Saadony M.T., Sitohy M.Z., Ramadan M.F., Saad A.M. (2021). Green nanotechnology for preserving and enriching yogurt with biologically available iron (II). Innov. Food Sci. Emerg. Technol..

[29] Rezende E.S.V., Lima G.C., Naves M.M.V. (2021). Dietary fibers as beneficial microbiota modulators: A proposed classification by prebiotic categories. Nutrition.

[30] Salehi M., Ghorbani M., Sadeghi Mahoonk A., Khomeiri M. (2021). Physicochemical, antioxidant and sensory properties of yogurt fortified with common purslane (*Portulaca oleracea*) extract. J. Food Meas. Charact..

[31] El-Sayed M., Awad S., Ibrahim A. (2019). Impact of purslane (*Portulaca oleracea* L.) extract as antioxidant and antimicrobial agent on overall quality and shelf life of Greek-style yoghurt. Egypt J. Food Sci..

[32] Obied W., Mohamoud E., Mohamed O. (2003). *Portulaca oleracea* (purslane): Nutritive composition and clinico-pathological effects on Nubian goats. Small Rumin. Res..

[33] Uddin M., Juraimi A.S., Hossain M.S., Nahar M., Un A., Ali M., Rahman M. (2014). Purslane weed (*Portulaca oleracea*): A prospective plant source of nutrition, omega-3 fatty acid, and antioxidant attributes. Sci. World J..

[34] de Souza P.G., Rosenthal A., Ayres E.M.M., Teodoro A.J. (2022). Potential functional food products and molecular mechanisms of *Portulaca oleracea* L. on Anticancer Activity: A Review. Oxidative Med. Cell. Longev..

[35] Petropoulos S.A., Fernandes Â., Dias M.I., Vasilakoglou I.B., Petrotos K., Barros L., Ferreira I.C. (2019). Nutritional value, chemical composition and cytotoxic properties of common purslane (*Portulaca oleracea* L.) in relation to harvesting stage and plant part. Antioxidants.

[36] Fernández-Poyatos M.d.P., Llorent-Martínez E.J., Ruiz-Medina A. (2021). Phytochemical composition and antioxidant activity of *Portulaca oleracea*: Influence of the steaming cooking process. Foods.

[37] Karoune S., Kechebar M.S.A., Douffi H., Djellouli A. (2017). Phenolic compounds and their antioxidant activities in *Portulaca oleracea* L. related to solvent extraction. Int. J. Biosci..

[38] Ranjah M.A. (2019). Lemongrass: A useful ingredient for functional foods. Int. J. Food Allied Sci..

[39] Sicari V., Loizzo M.R., Tundis R., Mincione A., Pellicano T.M. (2018). *Portulaca oleracea* L.(Purslane) extracts display antioxidant and hypoglycaemic effects. J. Appl. Bot. Food Qual..

[40] Montoya-García C.O., García-Mateos R., Becerra-Martínez E., Toledo-Aguilar R., Volke-Haller V.H., Magdaleno-Villar J.J. (2023). Bioactive compounds of purslane (*Portulaca oleracea* L.) according to the production system: A review. Sci. Hortic..

[41] Fukalova Fukalova T., García-Martínez M.D., Raigón M.D. (2022). Nutritional composition, bioactive compounds, and volatiles profile characterization of two edible undervalued plants: *Portulaca oleracea* L. and *Porophyllum ruderale* (Jacq.) Cass. Plants.

[42] Almashad A.A., Ibrahim Ramadan G.E., Abdelrazek R.H. (2019). Phytochemicals, antioxidant and volatile compounds evaluation of Egyptian purslane leaves. Arab. Univ. J. Agric. Sci..

[43] Carrasco F.R., Schmidt G., Romero A.L., Sartoretto J.L., Caparroz-Assef S.M., Bersani-Amado C.A., Cuman R.K.N. (2009). Immunomodulatory activity of *Zingiber officinale* Roscoe, *Salvia officinalis* L. and *Syzygium aromaticum* L. essential oils: Evidence for humor-and cell-mediated responses. J. Pharm. Pharmacol..

[44] Melilli M.G., Pagliaro A., Bognanni R., Scandurra S., Di Stefano V. (2020). Antioxidant activity and fatty acids quantification in Sicilian purslane germplasm. Nat. Prod. Res..

[45] Wang Z., He Z., Zhang D., Li H. (2021). Antioxidant activity of purslane extract and its inhibitory effect on the lipid and protein oxidation of rabbit meat patties during chilled storage. J. Sci. Food Agric..

[46] Farshori N.N., Al-Sheddi E.S., Al-Oqail M.M., Musarrat J., Al-Khedhairy A.A., Siddiqui M.A. (2014). Cytotoxicity assessments of *Portulaca oleracea* and *Petroselinum sativum* seed extracts on human hepatocellular carcinoma cells (HepG2). Asian Pac. J. Cancer Prev..

[47] Keser F., Karatepe M., Keser S., Tekİn S., Türkoğlu İ., Kaygİlİ O., Kirbag S. (2021). In vitro biological activities and phytochemical contents of *Portulaca oleracea* L.(Purslane). J. Phys. Chem. Funct. Mat..

[48] Seyedi Z., Amiri M.S., Mohammadzadeh V., Hashemzadeh A., Haddad-Mashadrizeh A., Mashreghi M., Qayoomian M., Hashemzadeh M.R., Simal-Gandara J., Taghavizadeh Yazdi M.E. (2023). Icariin: A promising natural product in biomedicine and tissue engineering. J. Funct. Biomater..

[49] Mousavi-Kouhi S.M., Beyk-Khormizi A., Amiri M.S., Mashreghi M., Hashemzadeh A., Mohammadzadeh V., Alavi F., Mottaghipisheh J., Sarafraz Ardakani M.R., Taghavizadeh Yazdi M.E. (2023). Plant gel-mediated synthesis of gold-coated nanoceria using ferula gummosa: Characterization and estimation of its cellular toxicity toward breast cancer cell lines. J. Funct. Biomater..

[50] Ghorani-Azam A., Mottaghipisheh J., Amiri M.S., Mashreghi M., Hashemzadeh A., Haddad-Mashadrizeh A., Nourbakhsh F., Nadaf M., Qayoomian M., Yazdi M.E.T. (2022). Resveratrol-mediated gold-nanoceria synthesis as green nanomedicine for phytotherapy of hepatocellular carcinoma. Front. Biosci..

[51] Nichani K., Li J., Suzuki M., Houston J.P. (2020). Evaluation of Caspase-3 activity during apoptosis with fluorescence lifetime-based cytometry measurements and phasor analyses. Cytom. Part A J. Int. Soc. Anal. Cytol..

[52] Yadav P., Yadav R., Jain S., Vaidya A. (2021). Caspase-3: A primary target for natural and synthetic compounds for cancer therapy. Chem. Biol. Drug Des..

[53] Tleubayeva M.I., Datkhayev U.M., Alimzhanova M., Ishmuratova M.Y., Korotetskaya N.V., Abdullabekova R.M., Flisyuk E.V., Gemejiyeva N.G. (2021). Component composition and antimicrobial activity of CO_2_ extract of *Portulaca oleracea*, growing in the Territory of Kazakhstan. Sci. World J..

[54] Seif M.M., Madboli A.-N., Marrez D.A., Aboulthana W.M.K. (2019). Hepato-Renal protective effects of egyptian purslane extract against experimental cadmium toxicity in rats with special emphasis on the functional and histopathological changes. Toxicol. Rep..

[55] Wang C., Liu Q., Ye F., Tang H., Xiong Y., Wu Y., Wang L., Feng X., Zhang S., Wan Y. (2021). Dietary purslane (*Portulaca oleracea* L.) promotes the growth performance of broilers by modulation of gut microbiota. AMB Express.

[56] Saleh S.R., Manaa A., Sheta E., Ghareeb D.A., Abd-Elmonem N.M. (2022). The synergetic effect of Egyptian *Portulaca oleracea* L. (Purslane) and *Cichorium intybus* L.(Chicory) extracts against glucocorticoid-induced testicular toxicity in rats through Attenuation of Oxidative Reactions and Autophagy. Antioxidants.

[57] Mousa A., Taha M., ELdeighdye S., Kamal A. (2023). The role of purslane in modulating diverse effects of high fat diet on biochemical, histological, and molecular parameters of rats’ liver. Braz. J. Biol..

[58] Nair G.G., Nair C.K.K. (2013). Radioprotective effects of gallic acid in mice. BioMed Res. Int..

[59] Saenthaweesuk S., Munkong N., Parklak W., Thaeomor A., Chaisakul J., Somparn N. (2017). Hepatoprotective and antioxidant effects of *Cymbopogon citratus* Stapf (Lemongrass) extract in paracetamolinduced hepatotoxicity in rats. Trop. J. Pharm. Res..

[60] Said A.M., Atwa S.A., Khalifa O.A. (2019). Ameliorating effect of gum arabic and lemongrass on chronic kidney disease induced experimentally in rats. Bull. Natl. Res. Cent..

[61] Burri S.C., Ekholm A., Håkansson Å., Tornberg E., Rumpunen K. (2017). Antioxidant capacity and major phenol compounds of horticultural plant materials not usually used. J. Funct. Foods.

[62] Oboh G., Puntel R., Rocha J. (2007). Hot pepper (*Capsicum annuum*, Tepin and *Capsicum chinese*, Habanero) prevents Fe^2+^-induced lipid peroxidation in brain–in vitro. Food Chem..

[63] Fouda F. (2022). Cytotoxicity assessments of *Portulaca oleracea* plant extracts on some cancer cells. Ann. Agric. Sci. Moshtohor.

[64] Valencia-Avilés E., García-Pérez M.E., Garnica-Romo M.G., de Dios Figueroa-Cárdenas J., Paciulli M., Martinez-Flores H.E. (2022). Chemical composition, physicochemical evaluation and sensory analysis of yogurt added with extract of polyphenolic compounds from *Quercus crassifolia* oak bark. Funct. Foods Health Dis..

[65] Nasser S. (2022). The addition of lemon peel powder affects the properties of yogurt. J. Food Dairy Sci..

[66] Sivakumar G. (2017). Effect of tulsi leaf extract on Physico-chemical and microbial quality of raw milk. Int. J. Sci. Environ. Technol..

[67] Saad A.M., Mohamed A.S., El-Saadony M.T., Sitohy M.Z. (2021). Palatable functional cucumber juices supplemented with polyphenols-rich herbal extracts. LWT-Food Sci. Technol..

[68] Fu Q., Zhou S., Yu M., Lu Y., He G., Huang X., Huang Y. (2022). *Portulaca oleracea* polysaccharides modulate intestinal microflora in aged rats in vitro. Front. Microbiol..

[69] Wang X., Kristo E., LaPointe G. (2019). The effect of apple pomace on the texture, rheology and microstructure of set type yogurt. Food Hydrocoll..

[70] Dello Staffolo M., Sato A.C., Cunha R. (2017). Utilization of plant dietary fibers to reinforce low-calorie dairy dessert structure. Food Bioprocess Technol..

[71] Osman D.M., Noureldin H.A., El-Gazzar F.E., Salman K.H. (2023). Fortification of ice milk with purslane (*Portulaca oleracea*) bioactive compounds. Assiut J. Agric. Sci..

[72] Ahmed I.A.M., Alqah H.A., Saleh A., Al-Juhaimi F.Y., Babiker E.E., Ghafoor K., Hassan A.B., Osman M.A., Fickak A. (2021). Physicochemical quality attributes and antioxidant properties of set-type yogurt fortified with argel (*Solenostemma argel* Hayne) leaf extract. LWT-Food Sci. Technol..

[73] Guemidi C., Saada D.A., Chabane O.A., Elmastas M., Erenler R., Yilmaz M.A., Tarhan A., Akkal S., Khelifi H. (2023). Antioxidant potential, lipid profile and sensory attributes of a functional yogurt formulated with hydroethanolic extract of *Mentha Piperita* L. Res. Sq..

[74] Cho W.-Y., Kim D.-H., Lee H.-J., Yeon S.-J., Lee C.-H. (2020). Journal of food quality evaluation of effect of extraction solvent on selected properties of olive leaf extract. J. Food Qual..

[75] Semeniuc C.A., Mandrioli M., Rodriguez-Estrada M.T., Muste S., Lercker G. (2016). Thiobarbituric acid reactive substances in flavored phytosterol-enriched drinking yogurts during storage: Formation and matrix interferences. Eur. Food Res. Technol..

[76] Alappat B., Alappat J. (2020). Anthocyanin pigments: Beyond aesthetics. Molecules.

[77] Li C., Liang J., Lin X., Xu H., Tadda M.A., Lan L., Liu D. (2019). Fast start-up strategies of MBBR for mariculture wastewater treatment. J. Environ. Manag..

[78] AOAC (2012). Official Methods of Analysis of AOAC International.

[79] Moczkowska M., Karp S., Niu Y., Kurek M.A. (2019). Enzymatic, enzymatic-ultrasonic and alkaline extraction of soluble dietary fibre from flaxseed–A physicochemical approach. Food Hydrocoll..

[80] Alamoudi S.A., Saad A.M., Alsubhi N.H., Alrefaei G.I., Al-Quwaie D.A., Binothman N., Aljadani M., Alharbi M., Alanazi H., Babalghith A.O. (2022). Upgrading the physiochemical and sensory quality of yogurt by incorporating polyphenol-enriched citrus pomaces with antioxidant, antimicrobial, and antitumor activities. Front. Nutr..

[81] Attard E. (2013). A rapid microtitre plate Folin-Ciocalteu method for the assessment of polyphenols. Open Life Sci..

[82] Herald T.J., Gadgil P., Tilley M. (2012). High-throughput micro plate assays for screening flavonoid content and DPPH-scavenging activity in *Sorghum bran* and flour. J. Sci. Food Agric..

[83] Politeo O., Jukić M., Miloš M. (2006). Chemical composition and antioxidant activity of essential oils of twelve spice plants. Croat. Chem. Acta.

[84] Li H., Wu X., Li X., Cao X., Li Y., Cao H., Men Y. (2021). Multistage extraction of star anise and black pepper derivatives for antibacterial, antioxidant, and anticancer activity. Front. Chem..

[85] Pino J.A., Barzola-Miranda S.E. (2020). Characterization of odor-active compounds in pechiche (*Vitex cymosa* Berteo ex Speng) fruit. J. Raw Mater. Proc. Foods.

[86] Jia N., Kong B., Liu Q., Diao X., Xia X. (2012). Antioxidant activity of black currant (*Ribes nigrum* L.) extract and its inhibitory effect on lipid and protein oxidation of pork patties during chilled storage. Meat Sci..

[87] Zhao R., Gao X., Cai Y., Shao X., Jia G., Huang Y., Qin X., Wang J., Zheng X. (2013). Antitumor activity of *Portulaca oleracea* L. polysaccharides against cervical carcinoma in vitro and in vivo. Carbohydr. Polym..

[88] Dai Z., Nair V., Khan M., Ciolino H.P. (2010). Pomegranate extract inhibits the proliferation and viability of MMTV-Wnt-1 mouse mammary cancer stem cells in vitro. Oncol. Rep..

[89] Ashour E.A., El-Hack M.E.A., Shafi M.E., Alghamdi W.Y., Taha A.E., Swelum A.A., Tufarelli V., Mulla Z.S., El-Ghareeb W.R., El-Saadony M.T. (2020). Impacts of green coffee powder supplementation on growth performance, carcass characteristics, blood indices, meat quality and gut microbial load in broilers. Agriculture.

[90] Schumann G., Klauke R. (2003). New IFCC reference procedures for the determination of catalytic activity concentrations of five enzymes in serum: Preliminary upper reference limits obtained in hospitalized subjects. Clin. Chim. Acta.

[91] Beutler E. (1994). G6PD deficiency. Blood.

[92] Bulut M., Selek S., Bez Y., Kaya M.C., Gunes M., Karababa F., Celik H., Savas H.A. (2013). Lipid peroxidation markers in adult attention deficit hyperactivity disorder: New findings for oxidative stress. Psychiatry Res..

[93] Armbruster D.A., Lambert P.A. (1996). Direct assay of LDL cholesterol: Comparing measurement and calculation. Lab. Med..

[94] Devi R., Sharma D. (2004). Hypolipidemic effect of different extracts of *Clerodendron colebrookianum* Walp in normal and high-fat diet fed rats. J. Ethnopharmacol..

[95] Dahlan H.A., Sani N.A. (2017). The interaction effect of mixing starter cultures on homemade natural yogurt’s pH and viscosity. Int. J. Food Stud..

[96] Korkmaz I.O., Bilici C., Korkmaz S. (2021). Sensory, pH, synaeresis, water-holding capacity, and microbiological changes in homemade yogurt prepared with maca (*Lepidium meyenii*) powder and propolis extract. Int. J. Gastron. Food Sci..

[97] Nguyen P.T., Kravchuk O., Bhandari B., Prakash S. (2017). Effect of different hydrocolloids on texture, rheology, tribology and sensory perception of texture and mouthfeel of low-fat pot-set yoghurt. Food Hydrocoll..

[98] Namir M., Iskander A., Alyamani A., Sayed-Ahmed E.T.A., Saad A.M., Elsahy K., El-Tarabily K.A., Conte-Junior C.A. (2022). Upgrading common wheat pasta by fiber-rich fraction of potato peel byproduct at different particle sizes: Effects on physicochemical, thermal, and sensory properties. Molecules.

[99] El-Saadony M.T., Khalil O.S.F., Osman A., Alshilawi M.S., Taha A.E., Aboelenin S.M., Shukry M., Saad A.M. (2021). Bioactive peptides supplemented raw buffalo milk: Biological activity, shelf life and quality properties during cold preservation. Saudi J. Biol. Sci..

