# Supplementary material for: Characterization of Portulaca oleracea Whole Plant: Evaluating Antioxidant, Anticancer, Antibacterial, and Antiviral Activities and Application as Quality Enhancer in Yogurt
Source: Molecules. 2023 Aug 3;28(15):5859. doi: 10.3390/molecules28155859 (PMC10421184; doi:10.3390/molecules28155859)
Supplement: Supplementary file 1 [file molecules-28-05859-s001.zip › molecules-2514385-supplementary.pdf]

Table S1. Growth parameters of Wister rats fed a diet supplemented with *P. oleracea* extract at three concentrations (50, 150, and 250) µg/g compared to BHA.

Group	Performance parameters		
	**IW (g)**	**FW (g)**	**BWG (g)**
Control.	247.5±0.5	307.1±0.4b	60.90±0.6b
BHA (250 µg/g)	246.5±0.2	298.3±0.3d	51.8±0.4d
PuE (50 µg/g)	247.1±0.1	305±0.9c	58.4±0.3c
PuE (150 µg/g)	247.5±0.3	311±0.5ab	64.6±0.6ab
PuE (250 µg/g)	246.9±0.4	315±0.2a	68.2±0.8a

Initial weight IW, Final weight FW, NC non-treated rats (negative control); PC rats fed BHA-supplemented diet (positive control); PuE-rats received a diet supplemented with PuE (50, 150, and 250 µg/g). The findings are presented as mean ± standard deviation; lowercase letters (a-d) in each column indicate significant differences between PuE concentration and BHA in rat diet using the LSD at p<0.05.

Table S2. color parameters and color change during the storage of yogurt samples for 30 days.

Yogurt samples	*L*		*a*		*b*		ΔE
	0d	30d	0d	30d	0d	30d	
Control	74.3±0.2^{b}	71.6±0.2^{a}	0.16±0.06^{a}	0.1±0.0^{a}	8.9±0.1^{d}	8.1±0.1^{c}	1.87±0.1^{a}
PuE 50	74.8±0.1^{b}	71.8±0.4^{b}	0.11±0.02^{ab}	0±0.0	9.3±0.2^{c}	8.5±0.4^{b}	1.45±0.2^{b}
PuE 150	75.2±0.6^{a}	72.6±0.6^{a}	0.09±0.001^{b}	0±0.0	10.6±0.8^{b}	9.3±0.8^{ab}	1.32±0.1^{c}
PuE 250	73.9±0.9^{c}	70±0.7^{c}	0.01±0.002^{c}	0±0.0	11.5±0.3^{a}	9.8±0.3^{a}	1.17±0.3^{d}

Lowercase letters (a-c) in each column indicate significant differences in color parameters in yogurt affected by *P. oleracea* extract concentrations during cold storage of 30 d at $p \leq 0.05$ using the LSD test. L, lightness (100) to darkness (0); (*a*) redness (+) to greenth (–); *b* yellowness (+) to blueness (–)

Table S3. The constituents of purslane extract-supplemented yogurt

Ingredients	YD (control)	PuYD1	PuYD2	PuYD3
Yogurt (g)	100	100	100	100
Sugar (g)	10	–*	–	–
PuE (mL)	–	50	50	50
Water (mL)	50	–	–	–

Control yogurt drink (YD), Yogurt drink supplemented PuE 50 µg/mL (PuYD1), Yogurt drink supplemented PuE 150 µg/mL (PuYD2), Yogurt drink enriched PuE 250 µg/mL (PuYD3).

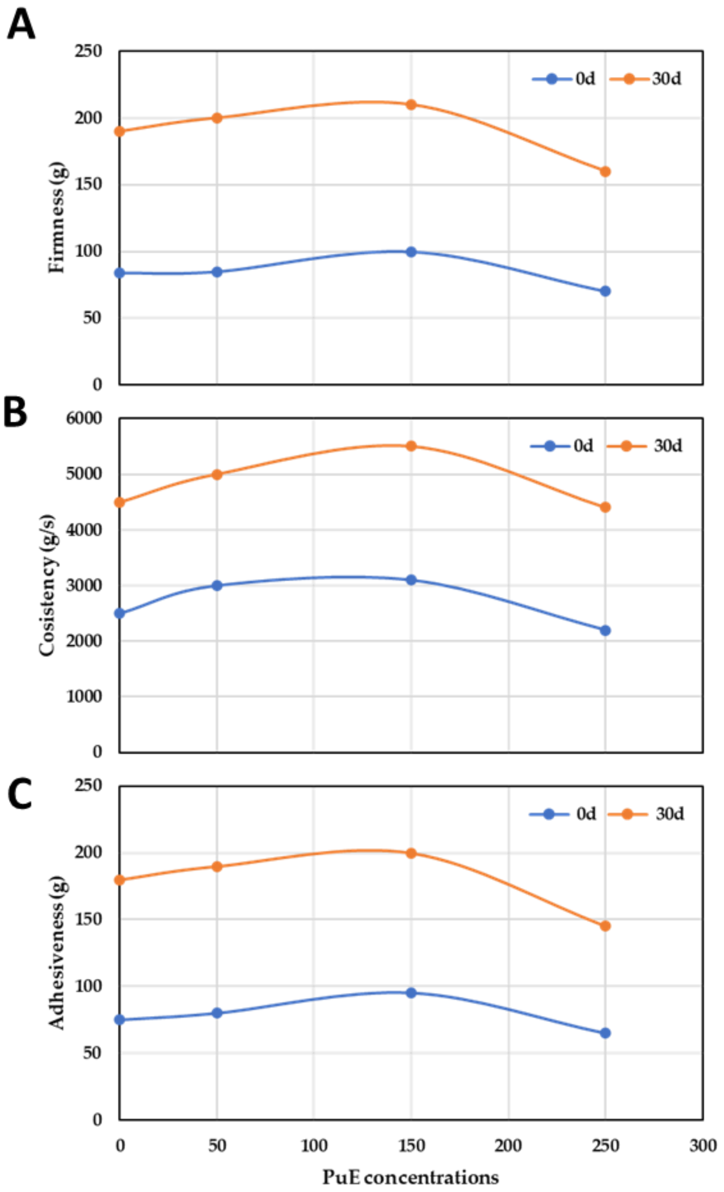

Figure S1. The impact of purslane extract at three concentrations (50, 150, and 250 μg/g) on yogurt texture properties (A, Firminess, B, consistency, C, adhesiveness) during cold storage.
